# Construction of crosstalk-free multi-functional phototherapeutic agents†

**DOI:** 10.1039/d4sc08796h

**Published:** 2025-02-12

**Authors:** Lixuan Dai, Wenxiu Li, Xiaoli Zhong, Mingguang Ren, Tony D. James, Weiying Lin

**Affiliations:** a Institute of Optical Materials and Chemical Biology, Guangxi Key Laboratory of Electrochemical Energy Materials, School of Chemistry and Chemical Engineering, Guangxi University Nanning Guangxi 530004 P. R. China weiyinglin2013@163.com; b Department of Chemistry, University of Bath Bath BA2 7AY UK chstdj@bath.ac.uk; c School of Chemistry and Chemical Engineering, Henan Normal University Xinxiang 453007 P. R. China; d China State Key Laboratory of Biobased Material and Green Papermaking, Key Laboratory of Pulp & Paper Science and Technology of Shandong Province/Ministry of Education, Qilu University of Technology (Shandong Academy of Sciences) Jinan 250353 P. R. China

## Abstract

Phototherapeutic diagnostics has attracted ever increasing interest due to its substantial promise within conventional cancer therapeutic paradigms. Consequently, the development of multi-functional phototherapeutic agents targeting specific organelles to uncover the close association of specific organelles with apoptotic signaling pathways is particularly appealing yet difficult to achieve. Here, we propose the concept of a crosstalk-free multi-functional phototherapeutic agent. This innovative phototherapeutic agent enables the concurrent delivery of highly efficient phototherapeutic treatment and crosstalk-free imaging, employing a dual-channel strategy. Differing from predecessors with single-channel multi-functional phototherapeutic functions, we engineered a dual-channel system to mitigate the competition between non-radiative and radiative relaxation processes, enabling both high fluorescence quantum yield and high photothermal conversion efficacy in one multi-functional phototherapeutic agent. The theranostic agent NIR-Cz was designed using this concept. Last but not least, using NIR-Cz, at a cellular level and *in vivo*, we observed a correlation between the average quantity of lipid droplets and the degree of apoptosis, which exhibited an increase with a non-monotonic trend and variable fluctuations. The concept of crosstalk-free multi-functional phototherapeutic agents is expected to provide a suite of powerful tools to elucidate the intricate relationships between organelles and apoptotic signaling pathways.

## Abstract 2

Phototherapeutic diagnostics has attracted ever increasing interest due to its substantial promise within conventional cancer therapeutic paradigms.

## Graphical abstract

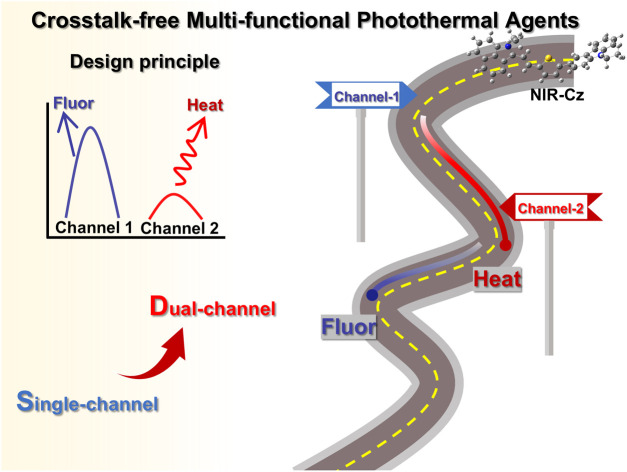

## Introduction

To counteract the threat to human life posed by cancer, a fatal disorder that has constantly plagued society, many cancer treatments have been developed, such as surgical resection, chemotherapy and radiation.^1–3^ Nevertheless, these traditional treatments have many disadvantages including being highly invasive, failing to completely remove tumors, and causing side effects associated with radiation.^4,5^ Thus, there is an urgent need to develop safer and effective strategies for cancer treatment. In recent years, phototherapy, including photothermal therapy (PTT) and photodynamic therapy (PDT), has attracted significant attention due to its potential to serve as an ideal treatment with minimal side effects and high selectivity.^6–9^ PTT is a non-invasive phototherapeutic technique that treats cancer by increasing the temperature of a tumor and is a highly selective and minimally invasive cancer treatment.^10,11^ In contrast to PDT, PTT is an oxygen-independent phototherapeutic strategy that can overcome the tumor hypoxic microenvironment to achieve tumor ablation and exhibits a strong effect for the treatment of deep tumors. Photothermal agents play an important role in thermal therapy, where they can act as a bridge to convert photons into heat inducing local heat shock, and have long been considered key to achieving effective cancer treatment.^12^

Organelles are functional parts of the cell, whose activity and chemical composition provide energy for the normal growth, repair, and proliferation of cells. Each organelle performs its specific role and cooperates with others to perform normal physiological functions. In recent years, researchers have found that many organelles play a key role in the process of apoptosis, actively participate in the regulation of related processes and act as “weathervanes”. Mitochondria have been reported to play a critical role in the activation of apoptosis, with the loss of mitochondrial membrane potential and the release of cytochrome c acting as key triggers for the activation of the caspase cascade. Likewise, lysosomes are also capable of directing apoptosis; the increase in lysosomal membrane permeability that occurs in the early stages of apoptosis results in the release of lysosomal enzymes, such as cathepsins, into the cytosol, which can subsequently induce or potentiate apoptotic processes.^13,14^ In recent years, phototherapeutic agents capable of specifically targeting organelles have emerged as a new research hotspot due to their ability to activate certain apoptotic signals more efficiently and specifically through precisely localized heat treatment.^15–17^ Unveiling new connections between organelles during the process of apoptosis induced by PTT which may help improve PTT and PDT efficacy.

To date, many excellent photothermal agents with targeting abilities for different organelles (*e.g.* nucleus, mitochondria, lysosomes, *etc.*) have been developed to guide the treatment of tumors. Feng *et al.* have developed a series of multi-functional phototherapeutic agents targeting the mitochondria by modulating the electron-donating properties of π-bridges, which exhibit efficient and balanced PDT and PTT performance, thereby achieving precise tumor phototherapy.^18^ Peng *et al.* have designed a multi-functional phototherapeutic agent, Scy, by substituting sulfur for oxygen atoms, which targets lysosomes and possesses the ability to resist viscosity interference, successfully achieving lysosome imaging at the cellular level and photothermal tumor ablation.^19^ However, conventional multi-functional phototherapeutic agents, which are designed to achieve both therapeutic and diagnostic purposes through a single channel, often result in suboptimal imaging performance. This, in turn, limits the application of multi-functional phototherapeutic agents in the study of organelles during the process of cellular apoptosis. Most conventional multi-functional phototherapies have a single excitation and absorption in the NIR (>700 nm) region, so they usually have a higher highest occupied molecular orbital (HOMO) and lower lowest unoccupied molecular orbital (LUMO) distribution. This smaller gap could enhance radiative processes and simultaneously suppress intersystem crossing (ISC)-induced energy loss. The conventional strategy of using a single channel for building multi-functional phototherapeutic agents with the capability of imaging and phototherapy further enhances the competition between radiative and non-radiative relaxation (Table S1†). Therefore, the development of multi-functional phototherapeutic agents capable of simultaneously achieving high spatiotemporal resolution, high fluorescence quantum yield and high photothermal conversion efficacy or high photodynamic efficiency remains a significant challenge.

**Scheme 1:**
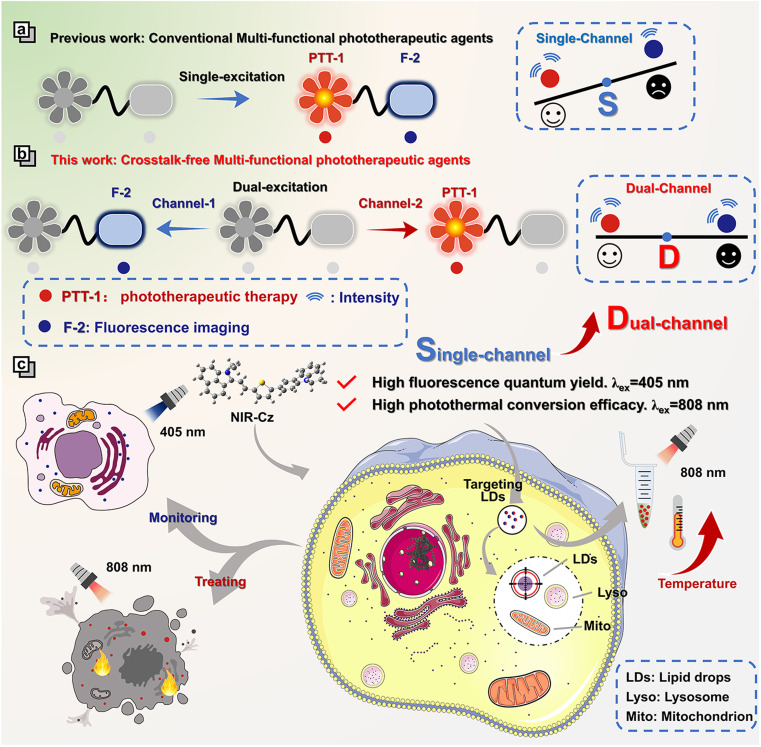
Schematic illustration of (a) conventional multi-functional phototherapeutic agents and (b) crosstalk-free multi-functional phototherapeutic agents NIR-Cz for (c) PTT and monitoring lipid droplets in real time.

Herein, we present a pioneering concept: crosstalk-free multi-functional phototherapeutic agents based on a dual-channel strategy (Scheme 1). This new type of multi-functional phototherapeutic agent mitigates the competition between radiative and non-radiative relaxation existing for single-channel systems, thereby enabling the integration of high resolution spatiotemporal imaging and high therapeutic efficiency into one phototherapeutic agent. As a proof of concept, we developed a theranostic agent, NIR-Cz, characterized by two distinct and mutually independent channels, which enables both high fluorescence quantum yield and high photothermal conversion efficiency using two separate channels (channel 1 and channel 2). Our investigation into the organelle-targeting and photothermal properties of NIR-Cz revealed that the theranostic agent specifically targets lipid droplets (LDs) and could trace the LD distribution, while efficiently enabling excellent photothermal therapy when using channel 2. For the first time, integrating high fluorescence quantum yield and high photothermal conversion efficiency into one phototherapeutic agent through a dual-channel strategy enables accurate crosstalk-free imaging for an organelle dynamic study during PTT-induced apoptosis. We anticipate that this research could provide an innovative strategy for the development of multi-functional phototherapeutic agents coupled with both high resolution spatiotemporal imaging and high therapeutic efficiency. Such multi-functional phototherapeutic agents exhibit significant potential for elucidating the mechanisms exhibited by organelles during apoptosis and for guiding the development of next generation multi-functional phototherapeutic agents.

## Experimental section and methods

### Materials

All reagents and solvents used in the experimental procedure were purchased and used directly without further purification. General chemicals and inorganic chemicals (various analytes) were purchased from Energy Chemical (Shanghai, China). All organic solvents were obtained from Sinopharm Chemical Reagent Company (Shanghai, China).

### Instruments

All nuclear magnetic resonance (NMR) spectra including (^1^H NMR) and (^13^C NMR) were obtained using an AVANCE III 400 MHz Digital NMR Spectrometer, with TMS as an internal standard. High-resolution mass spectrometric (HRMS) analyses were performed on a Finnigan MAT 95 XP spectrometer. Absorption spectra and fluorescence spectra were obtained on a Shimadzu UV-2700 Power spectrometer and HITACHI F4700 fluorescence spectrophotometer, respectively. The pH of phosphate buffered saline (PBS) buffer solutions was calibrated using a PHS-3E digital pH meter (LEICI, Shanghai, China). Confocal luminescence imaging was carried out using a Leica TCSSP8 DIVE laser scanning confocal microscope (Leica, GER). Photothermal images were obtained using an NIR thermal imager HM-TPH10-3AUF and HM-TPH21Pro-3AQF (Hikvision, Hangzhou, China).

### Synthesis and characterization

The full synthetic routes of the target compound and intermediates are provided in the ESI.†

### Statistical analysis

The results are expressed as the mean ± standard error. Statistical comparisons between two groups were performed using the two-tailed, unpaired student's *t*-test. Statistical significance is indicated as *P < 0.05, **P < 0.01, or ***P < 0.001. Here, *P < 0.05 is considered as significant, **P < 0.01 is considered as very significant, and ***P < 0.001 is considered as extremely significant.

## Results and discussion

### Design and synthesis of NIR-Cz

To achieve high spatiotemporal resolution imaging and implement phototherapeutic therapy using one multi-functional phototherapeutic agent, here we outline three key requirements. First, an ideal multi-functional phototherapeutic agent with both imaging and therapy capacity should possess a high fluorescence quantum yield, which contributes to enhanced imaging sensitivity, resolution, signal-to-noise ratio, and accuracy. Second, it is essential to possess good organelle targeting specificity to support precisely localized treatment. Third, multi-functional phototherapeutic agents should have desirable photothermal conversion efficiency or high photodynamic efficiency to achieve tumor ablation.

**Fig. 1:**
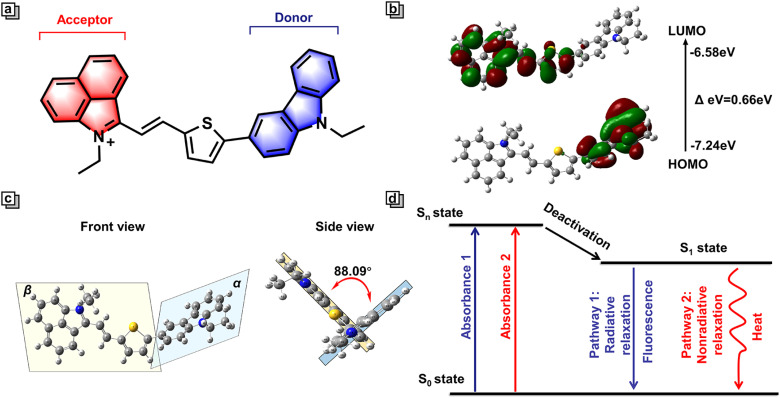
(a) Structure of NIR-Cz. (b) HOMO/LUMO of NIR-Cz in the excited state at the TD-DFT B3LYP/6-311G level. (c) Front view and side view of the geometry-optimized configuration of NIR-Cz. (d) Schematic illustration of two types of transitions in NIR-Cz under photoexcitation.

To design multi-functional phototherapeutic agents that meet these criteria, we focused our attention on dual-channel organic donor (D)–π–acceptor (A) D–π–A molecules with intramolecular charge transfer (ICT). Research suggests that D–π–A molecules are widely utilized for the construction of high quality photothermal agents. Indeed, as a classical D–π–A structure, NIR-Cz consists of two parts, including a carbazole unit and a naphtholactam cation unit. As an electron donor, the carbazole unit is an optical material with excellent stability and bright photophysical properties (Fig. 1a).^20^ We found that the incorporation of carbazole into fluorescent dyes may enhance their ability to exhibit dual-channel imaging capabilities, hence we used carbazole as the D in NIR-Cz.^21,22^ Moreover, the introduction of carbazole effectively reduces the LUMO energy and thus enhances the photostability of the blue channel.^23^ Through the calculation of the energy levels for the HOMO and LUMO, we conducted an in-depth analysis of the ICT system for NIR-Cz. Specifically, we calculated the energy levels of the HOMO (−7.24 eV) and LUMO (−6.58 eV) in the S1 state (Fig. 1b). The distribution of the HOMO electrons was primarily concentrated on the carbazole unit, while the LUMO was primarily spread out over the naphtholactam cation unit, thus indicating the successful construction of an ICT system. Additionally, the introduction of thiophene to produce a highly twisted structure not only leads to a large redshift, but also enables the possibility of generating two independent channels. Theoretically, time-dependent density functional theory (TD-DFT) supported a highly twisted structure within the molecule. As shown in Fig. 1c, the dihedral angle between the donor plane (*α*) and the acceptor plane (*β*) was calculated to be 88.09° (*θ*_*αβ*_). Moreover, the center of the twisted structure is located at the junction of the carbazole and cationic parts, which effectively reduces the degree of conjugation between the donor and acceptor units, allowing the fluorescence of the carbazole part to be released independently. Meanwhile, the twisting geometry of NIR-Cz due to the strong electron push–pull system allows for a certain amount of orbital mixing, which favours active intramolecular motion and contributes to non-radiative relaxation, ultimately increasing the photothermal efficiency.^24^ In addition, in order to target lipid droplets, an ethyl was introduced at the 9-position of carbazole to enhance the lipophilicity. To summarize, NIR-Cz was endowed with dual-channels, which are radiative-enhanced and non-radiative-enhanced relaxation, respectively. Furthermore, the clever design provides a strategy based on dual-channels to achieve high quantum yield and superior photothermal conversion efficiency simultaneously (Fig. 1d).

NIR-Cz, obtained by the Knoevenagel condensation reaction catalyzed by glacial acetic acid, appears as a blue-green colored powder, in a yield of approximately 65%. The structures of NIR-Cz and intermediates were verified by HR-MS, ^1^H NMR and ^13^C NMR (Fig. S22–S26†).

### Photophysical properties of channel 1 *in vitro*

**Fig. 2:**
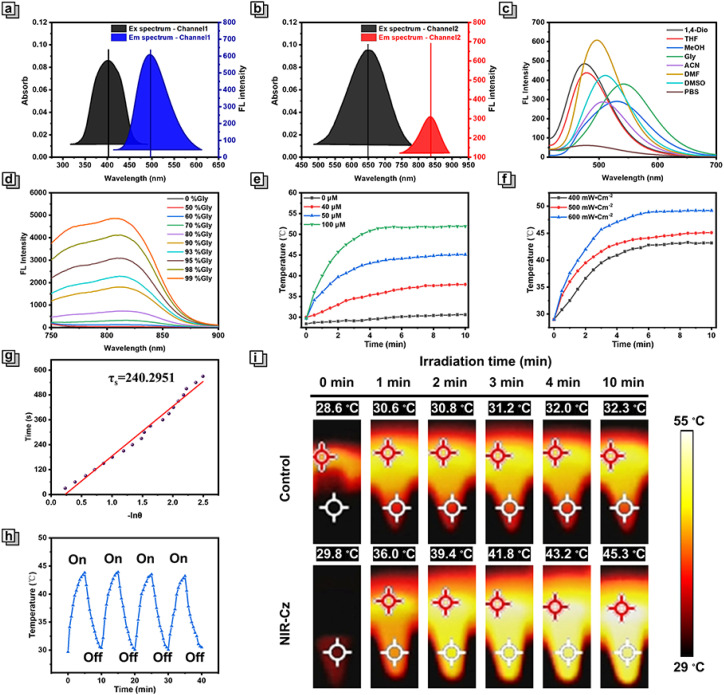
(a) Excitation and emission spectra of NIR-Cz in DMF (channel 1: excited at 405 nm). (b) Excitation and emission spectra of NIR-Cz in DMF (channel 2: excited at 690 nm). (c) Emission spectra of NIR-Cz in various solvents (*λ*_ex_ = 405 nm). (d) Emission spectra of NIR-Cz in PBS with various viscosities: glycerol (*λ*_ex_ = 690 nm). (e) Photothermal heating curves of NIR-Cz in PBS with various concentrations (0, 40, 50, and 100 μM) under an 808 nm laser (500 mW cm^−2^ and 10 min). (f) Photothermal heating curves of NIR-Cz in PBS (50 μM) under different laser intensities (400, 500, and 600 mW cm^−2^) for 10 min. (g) Plot of time *versus* −ln *θ* of NIR-Cz (the data from the cooling curve). (h) Photothermal stability study of NIR-Cz (50 μM, PBS) during four cycles of heating–cooling processes. (i) Infrared thermographs of NIR-Cz irradiated with an 808 nm laser (500 mW cm^−2^, 50 μM, and 10 min), with the temperature measured at the fixed white cursor.

After identifying a dual-channel approach as an appropriate solution for combined imaging and PTT, we set out to verify our design idea. First, the optical properties of NIR-Cz were assessed *via* UV-vis absorption and fluorescence emission spectra (Fig. S1†). In the UV-vis absorption spectra, NIR-Cz possesses two characteristic absorption peaks in DMF, achieving dual-band absorption with a smaller peak at 405 nm and a maximum at 660 nm (Fig. 2a and b), termed channel 1 and channel 2, respectively. Immediately afterwards, the emission spectra of these two characteristic peaks were obtained separately in a variety of solvents. The fluorescence behaviour of channel 1 was evaluated first; channel 1 exhibited an emission peak in the range of 490 nm to 540 nm across a series of solvents, all of which exhibited good fluorescence intensity and high fluorescence quantum yield (Fig. 2c and Table S2†). It is worth noting that there is no significant change in the maximum fluorescence intensity of the theranostic agent in MeOH and glycerol, and thus the impact of viscosity and polarity on the fluorescence intensity of NIR-Cz can be ignored. To explore the applicability of NIR-Cz for various applications, the fluorescence stability and selectivity of NIR-Cz were investigated. The results indicate that oxidizing ROS and RNS, reducing RSS, and common amino acids do not interfere with the signals, which remain “turned-on” (Fig. S2†). The excellent photophysical properties of the carbazole unit offer a strong foundation for the photostability of NIR-Cz, with the fluorescence residual rate of the sensor remaining close to 1.0 after up to 1 h of continuous excitation irradiation with a 405 nm laser (Fig. S3†). The above results confirm that channel 1 of NIR-Cz exhibits excellent performance.

### Photophysical and photothermal properties of channel 2 *in vitro*

Then we evaluated channel 2 emission characteristics, and the excitation wavelength was adjusted to the optimal excitation wavelength. At first, NIR-Cz exhibited a negligible emission peak in the range of ∼750–900 nm. After further increasing the excitation energy (increasing the voltage), the intensity and wavelength of the emission peak remained essentially the same as before in most solutions, except for glycerol (Fig. S4†). As such, the optical properties of the multi-functional phototherapeutic agent in PBS/glycerol mixtures with different glycerol fractions were determined. The fluorescence intensity of NIR-Cz exhibited a good viscosity dependence (Fig. S5†), with the intensity at 808 nm increasing with increasing glycerol fraction and reaching a maximum at 99% glycerol (with an ultra-low fluorescence quantum yield of approximately 0.0006%) (Fig. 2d), up to about 231-fold of that in PBS. Notably, when the glycerol content is below 50%, the fluorescence intensity of NIR-Cz is almost constant. For normal cells, the viscosity is in the range of 1–2 cP in the cellular cytoplasm, which is is far lower than the viscosity in solutions containing 50% glycerol.^25,26^NIR-Cz excited by 660 nm also exhibited good pH stability (Fig. S6†). In summary, the fluorescence intensity of NIR-Cz excited by 660 nm is viscosity dependent (high viscosity) and not polarity dependent. These results indicate that NIR-Cz exhibits potential as a photothermal agent excited by long wavelengths, due to the relaxation of the long wavelength excited state being principally through nonradiative pathways; as such, we subsequently evaluated its photothermal properties.

To verify the potential for photothermal therapy of NIR-Cz under NIR laser irradiation, the photothermal conversion at different concentrations in PBS was evaluated. When mixtures were irradiated using an 808 nm NIR laser (500 mW cm^−2^) for 10 min, all solutions exhibited a significant increase in temperature, which was positively correlated with the multi-functional phototherapeutic agent concentration (Fig. 2e and i). In contrast, there was no significant change in the temperature for the group without NIR-Cz. Among them, when the NIR-Cz concentration was 50 μM, Δ*T* (temperature change) was approximately 15 °C after 5 min of irradiation and rose to 23 °C when the concentration was increased to 100 μM. Considering the biocompatibility and photothermal efficiency of NIR-Cz, the effect of laser irradiation power for increasing temperature using a 50 μM photothermal agent was investigated (Fig. 2f and g). These results indicated that the photothermal effect of the multi-functional phototherapeutic agent is strongly related to the irradiation time, the irradiation laser power and the photothermal agent concentration. The excellent performance of the photothermal agent during the four cycles of the heating and cooling process confirmed the good photothermal stability of NIR-Cz (Fig. 2h). Furthermore, the photothermal conversion efficiency (PCE) of NIR-Cz was calculated to be 35.8% irradiated with an 808 nm laser, which is higher than that of conventional photothermal agents such as indocyanine green (ICG) (13.7%) (Fig. 2g, S7 and S8†).

### Fluorescence properties of channel 1 in cells

**Fig. 3:**
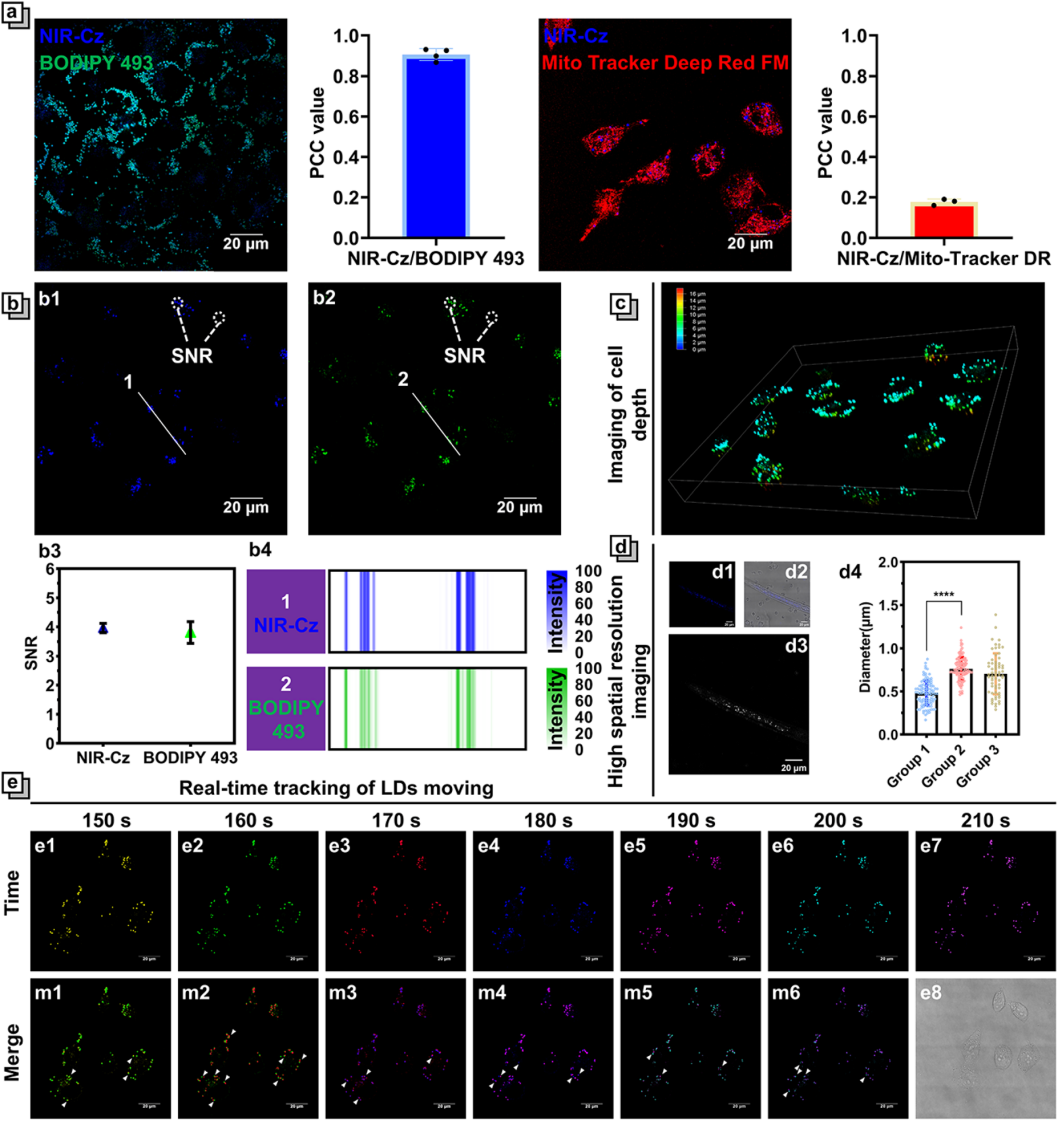
Fluorescence properties of channel 1. (a) Confocal fluorescence images of HeLa cells co-stained with NIR-Cz (10 μM, *λ*_ex_ = 405 nm, and *λ*_em_ = 450–530 nm) and commercial dyes for organelles, including BODIPY 493/503 (10 μM, *λ*_ex_ = 488 nm, and *λ*_em_ = 498–530 nm) and Mito-Tracker Deep Red FM (2 μM, *λ*_ex_ = 644 nm, and *λ*_em_ = 654–690 nm), and illustrations (right side) of the quantitative analysis of NIR-Cz and commercial dye colocalization; data are mean ± SEM (*n* = 3). (b) Comparison of signal-to-noise ratios (SNRs) (b3) between NIR-Cz (b1, 10 μM, *λ*_ex_ = 405 nm, and *λ*_em_ = 450–530 nm) and BODIPY 493/503 (b2, 10 μM, *λ*_ex_ = 488 nm, and *λ*_em_ = 498–530 nm), and b4 shows fluorescence intensity profiles along white solid lines 1 and 2. (c) Front view of 3D confocal fluorescence images of HeLa cells stained with NIR-Cz. The pseudocolors represent depth, with colors ranging from dark blue (bottom) to light blue, green, yellow, and red (top). (10 μM, *λ*_ex_ = 405 nm, and *λ*_em_ = 450–530 nm). (d) Confocal fluorescence images of *C. elegans* depicting the (d1) bright field, (d2) blue fluorescence channel, and (d3) grayscale fluorescence channel, and (d4) quantitative analysis of LD diameters (group 1: HeLa cells stained with NIR-Cz; group 2: HeLa cells pre-incubated with OA for 4 hours before being stained with NIR-Cz; group 3: *C. elegans* stained with NIR-Cz). (e). Confocal fluorescence of HeLa cells stained with NIR-Cz (10 μM, *λ*_ex_ = 405 nm, and *λ*_em_ = 450–530 nm). Different pseudocolors are used to illustrate the fluorescence images at different times (e1–e7). Bright-field image (e8). (m1–m6) Merges of images at 150/160 s (m1), 160/170 s (m2), 170/180 min (m3), 180/190 min (m4), 190/200 min (m5), and 200/210 min (m6). LDs exhibiting significant movement are marked with white arrows. Scale bar: 20 μm.

The superior multifunctionality of NIR-Cz for PTT and fluorescence emission was demonstrated by its excellent performance during *in vitro* experiments; as such, we used the multi-functional phototherapeutic agent for *in vitro* fluorescence imaging and photothermal studies in cells. First, the fluorescence properties of NIR-Cz were investigated using confocal microscopy (Fig. S9†). The bright fluorescence from the theranostic agent was observed upon excitation using a 405 nm laser, while no significant fluorescence could be observed upon excitation using a 660 nm laser due to the low-viscosity environment of cells. This result is consistent with the previous findings on spectral properties *in vitro*. We then carefully investigated the properties of NIR-Cz excited with a 405 nm laser and confirmed its ability as a multi-functional phototherapeutic agent to target LDs in HeLa cells. Considering the potential targeting properties of NIR-Cz for lipids and negative charges, LDs and mitochondria were chosen as target organelles for evaluation. BODIPY 493, Nile red and Mito-Tracker Deep Red FM were selected as references for LD and mitochondria localization, respectively. All commercial dyes were incubated separately with cells treated with oleic acid at 37 °C for 30 min, and NIR-Cz was then added to the imaging dish and incubated for an additional 30 min, followed by two washings with PBS before imaging. Pearson's colocalization coefficient between NIR-Cz and BODIPY 493 was 0.90 (between NIR-Cz and Nile red, it was 0.86, Fig. S11†), and much higher than 0.18 observed for NIR-Cz and Mito-Tracker Deep Red FM (Fig. 3a). Interestingly, unlike conventional hemicyanine dyes, NIR-Cz primarily accumulates in lipid droplets rather than mitochondria. This phenomenon is likely attributed to the greater lipophilicity of the theranostic agent compared to its affinity for the negative membrane potential of the mitochondrial inner membrane.

The log *P* value is commonly employed as a criterion for assessing the lipophilicity of small molecular compounds. It represents the logarithm of the distribution ratio of a compound between a polar solvent (typically water) and a non-polar solvent (such as octanol). A high log *P* value indicates strong lipophilicity, suggesting a preference for dissolution in non-polar solvents. To further elucidate NIR-Cz targeting specificity towards LDs, ChemDraw and two other computational models were utilized to calculate the Clog *P* (calculated log *P*) of NIR-Cz (Table S3†). Using ChemDraw, we calculated the Clog *P* of NIR-Cz to be 10.58, while even in XLOGP3 and Molinspiration models,^27^ the value exceeded 8, confirming that NIR-Cz has strong hydrophobicity. To validate the effectiveness of using Clog *P* values to determine the lipophilic targeting of probes, several organelle commercial dyes were selected and their values were calculated (Table S2†). The results indicated that the Clog *P* values for mitochondrial dyes were significantly lower than those for LD dyes, and the values for lipid droplet dyes generally ranged between 7 and 10. This theoretical result aligns well with our experimental findings.

An important parameter to describe the quality of imaging is the signal-to-noise ratio (SNR), and it is worth mentioning that NIR-Cz has a satisfactory SNR of not less than 4 during imaging and is almost comparable to that of the commercial dye BODIPY 493 at the same concentration (Fig. 3b). NIR-Cz not only offers excellent imaging quality, but also excellent light stability, enabling high-quality imaging over long periods of time (Fig. S10†). Also, thanks to the excellent targeting capability and high SNR of NIR-Cz, high-quality 3D depth imaging maps of the LDs of living cells were obtained *via* 3D patterning, where the different false-color scales ranging from dark blue, green, and yellow to red represent increasing depth (Fig. 3c and S12†).

LDs are small organelles, typically reported to have diameters less than 1 μm under normal conditions. Therefore, accurately quantifying the diameter of LDs in cells is a crucial criterion for evaluating whether an agent possesses high resolution spatial imaging capabilities. In this study, HeLa cells were divided into two groups: group 1 was directly treated with NIR-Cz, while group 2 was pre-incubated with oleic acid (OA) for 4 hours before the addition of NIR-Cz (Fig. S13†). After performing confocal imaging using channel 1, we conducted a quantitative analysis of the diameters of LDs within the cells. The results revealed that the diameter of LDs in the untreated HeLa cells was 0.48, whereas after OA treatment, the diameter increased to 0.75 (Fig. 3d). This change is attributed to oleic acid-induced lipid accumulation within the cells, which subsequently led to an increase in both the number and size of the LDs.

LDs are a kind of highly active organelle; therefore, monitoring the movement of LDs in real time is of great significance. Based on its good fluorescence properties, NIR-Cz should be able to track LDs moving with high temporal and spatial resolution. HeLa cells were incubated with 10 μM NIR-Cz for 30 min; image acquisition was performed continuously for 10 min in temporal imaging mode, and the time interval was set to 10 s per frame. Among them, the fluorescence color was replaced with a pseudocolor, and each image captured from a different time is denoted by a different pseudocolor for comparison purposes (Fig. 3d). The relative motion of the LDs can then be clearly observed after the merging of images from adjacent epochs. These results confirmed the powerful real-time high spatial resolution imaging capability of NIR-Cz under illumination of a 405 nm laser.

### Fluorescence properties of channel 1 *in vivo*

Building on the results confirming the ability of NIR-Cz to support high resolution spatial imaging of LDs in cells, we further assessed whether this capability could be extended to *in vivo* applications. *Caenorhabditis elegans* (*C. elegans*), a well-established model organism with advantageous biological characteristics such as small size and excellent transparency, was chosen for this study.^28^ The biological properties of *C. elegans* also support the extrapolation of our findings to other biological contexts. The experimental results indicated that following NIR-Cz wash-free staining, LDs within *C. elegans* were clearly observable in the blue channel (Fig. 3d). Quantitative analysis of the diameters of these LDs indicated that the average diameter of LDs in *C. elegans* is approximately 0.70 μm, similar to the diameter of LDs in untreated HeLa cells. Subsequently, we performed 3D pattern imaging of *C. elegans*, further supporting the conclusion that NIR-Cz is applicable for *in vivo* imaging (Fig. S14†). Additionally, the results confirmed that NIR-Cz provided a SNR value consistent with our cell experiments for the imaging of *C. elegans* (Fig. S15†). These findings confirm that NIR-Cz can be effectively utilized for labeling and analyzing LDs in *C. elegans*, making it suitable for *in vivo* studies.

### Photothermal properties of channel 2 in cells

**Fig. 4:**
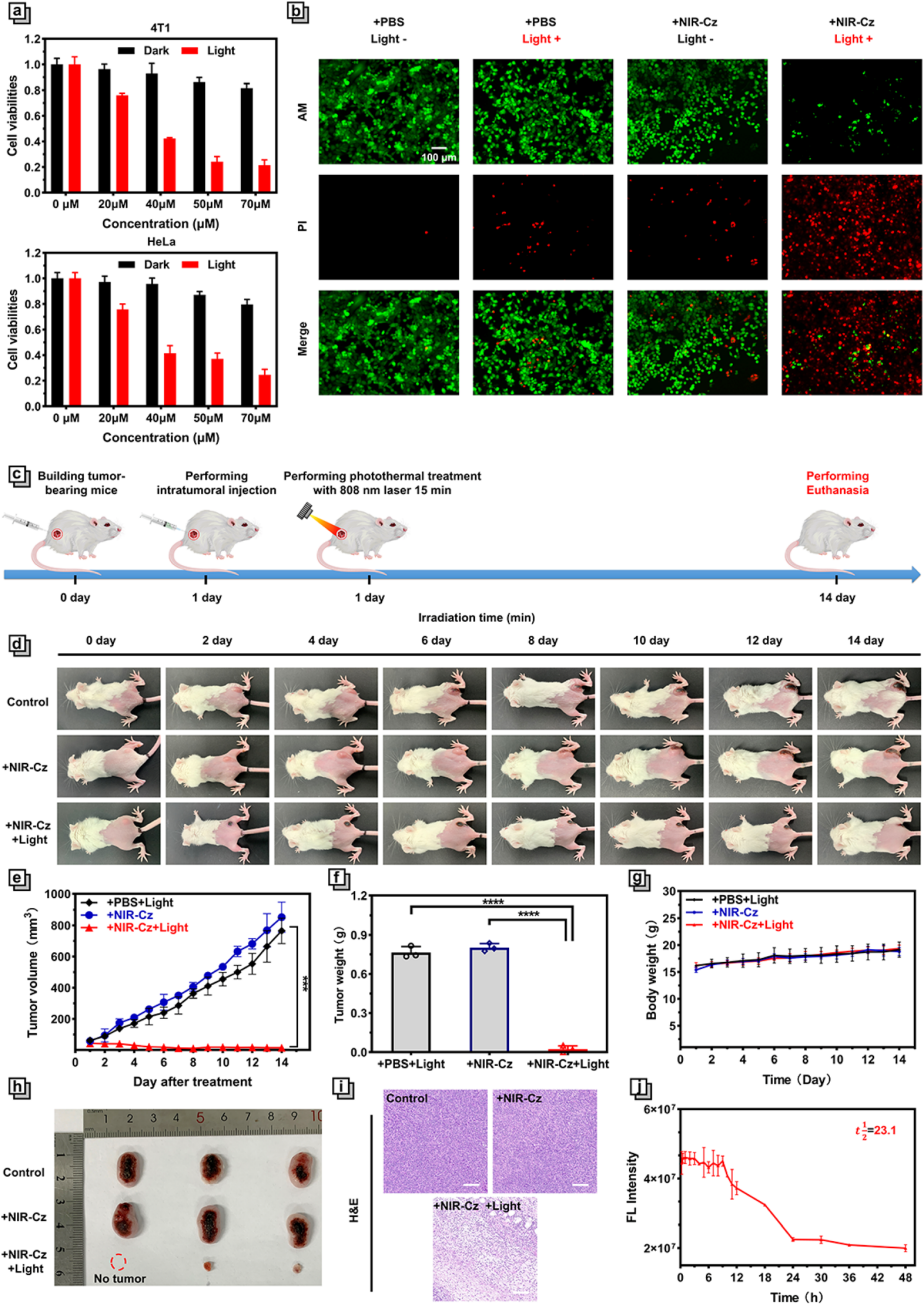
Photothermal properties of channel 2. (a) Cell viability of HeLa and 4T1 cells incubated with NIR-Cz at various concentrations in the dark and after 808 nm light irradiation (500 mW cm^−2^ and 5 min). (b) Calcein-AM (green) (*λ*_ex_ = 488 nm and *λ*_em_ = 500–580 nm) and propidium iodide (red) (*λ*_ex_ = 535 nm and *λ*_em_ = 600–625 nm) co-staining fluorescence images of 4T-1 cells after different treatments. 808 nm laser irradiation (500 mW cm^−2^ and 5 min) was conducted after cells were incubated with NIR-Cz (50 μM). Scale bar: 100 μm. (c) Schematic illustration of the *in vivo* experiment timeline. (d) Digital photographs of mice during various treatments. (e) Relative tumor volume changes of mice during various treatments. (f) Average tumor weights after various treatments. (g) Relative body weight changes of the mice during various treatments (*n* = 3). (h) Digital photograph of mice tumor after various treatments. (i) Histological H&E analysis of tumor tissues after various treatments. Scale bar: 100 μm. (j) Fluorescence intensity in tumor from mice post-administration of NIR-Cz at various time intervals.

Encouraged by the positive photothermal performance of NIR-Cz*in vitro*, we next investigated the phototherapeutic application of the multi-functional photothermal agent at the cellular level. The toxicity of NIR-Cz was investigated using an MTT assay in two types of normal cells: 3T3 cells and Cos7 cells. The results indicated that the probe did not exhibit significant toxicity (Fig. S16†). Two types of cancer cells of human and murine origin, respectively, HeLa cells and 4T-1 cells, were then selected for estimating phototoxicity and dark toxicity using the MTT method. The two groups of identical cells were divided into a phototoxicity group and a dark toxicity group and cultured on a 96-well plate. Among them, the cells in the phototoxicity group were exposed to 808 nm laser (500 mW cm^−2^) irradiation for 5 min, and the cells in the dark toxicity group were carefully protected. As described in the results, NIR-Cz exhibited negligible toxicity in the dark toxicity group in either cell type, with over 90% cell viability up to a concentration of 50 μM being observed (Fig. 4a). However, the cells of the phototoxicity group exhibited a lower survival rate that correlated with the multi-functional photothermal agent's concentration, and all survival rates were significantly lower than those of the cells in the unprocessed group, dropping to less than 30% at a concentration of 50 μM. These results indicated that NIR-Cz has excellent PTT efficacy and exhibited reliable biosafety at appropriate concentrations.

Next, we examined the PTT efficacy of NIR-Cz*via* confocal imaging combined with live/dead cell staining. It is well known that calcein-AM, a live cell dye that easily penetrates live cell membranes and is triggered by esterases, specifically labels live cells with green fluorescence, whereas propidium iodide only penetrates broken cell membranes and thus stains dead cells with red fluorescence. As shown in Fig. 4b, the cells in the first three groups are almost all labeled with green fluorescence, *i.e.*NIR-Cz or just 808 nm laser irradiation does not cause apoptosis. Only cells incubated with a 50 μM theranostic agent and irradiated with an 808 nm laser exhibit significant PTT effects. These results confirm the excellent killing effect of NIR-Cz on cancer cells under 808 nm laser irradiation (see also the high-magnification image in Fig. S17†).

In view of the excellent performance of NIR-Cz*in vitro*, *in vivo* photothermal tumor ablation was evaluated (Fig. 4c). As such, the photothermal effect of NIR-Cz irradiated using an 808 nm laser was monitored in xeno-graft 4T1 tumor-bearing BALB/c mice. When the tumor volume reached ≈60 mm^3^, all tumor-bearing mice were randomly divided into three groups: control group (mice injected with PBS and then irradiated with an 808 nm laser (500 mW cm^−2^) only once during the process of photothermal therapy, for a duration of 15 min); + NIR-Cz group (mice injected with the phototherapeutic agent (PBS) only); + NIR-Cz and + light group (mice injection with the phototherapeutic agent (PBS) and then irradiated with an 808 nm laser).

A thermal imaging camera was used to monitor the tumor temperature in each group of mice during the process of *in vivo* tumor ablation experiments (Fig. S18†). In the control group, the temperature at the tumor sites exhibited only a small change when the mice were only exposed to 808 nm laser irradiation without pretreatment with NIR-Cz. However, under the same irradiation conditions, the temperature at the tumor site rapidly increased to 44 °C for the mice of the “+ NIR-Cz and + light group”. Meanwhile, the maximum temperature did not exceed 50 °C even after more than 10 min of irradiation, reducing accidental damage to normal tissues, demonstrating that NIR-Cz could support low-temperature photothermal treatment. After photothermal treatment, the health of the mice was meticulously monitored, and the tumor volume, body weight and digital photographs were collected every two days for 14 days to assess the effect of the low-temperature photothermal treatment (Fig. 4d–g). The results are illustrated in Fig. 4d and c; the tumor growth is inhibited significantly in the “+ NIR-Cz and + light group”, where in one case complete tumor ablation was observed (Fig. 4h), while the tumor of mice from the other two groups grew rapidly. Next, we performed a power analysis using the Two-Sample *t*-test feature of Origin software. The results, presented in Table S3,† reveal that due to the significant therapeutic effects, for a sample size of three per group, the power value reaches 1, which fulfils the experimental requirements. All mice were euthanised 14 days after treatment and the tumors were collected from the different groups to evaluate the pathology after hematoxylin/eosin (H&E) staining. Morphological observation indicated that the cells in the tumor tissue were neatly arranged, while the cells in the control group were plump with a clearly visible nucleus and cytoplasm. Conversely, cells in the treatment group exhibited disorganized arrangement, accompanied by cytoplasmic shedding and other signs of severe damage, as well as prominent features of necrosis (Fig. 4i). These experiments confirmed the good photothermal therapeutic effect of NIR-Cz under 808 nm excitation.

After intratumoral injection was performed, we investigated the circulation half-life of NIR-Cz in the mice by collecting fluorescence signals at different times following intertumoral injection *via* a small animal *in vivo* imaging system. As shown in Fig. S19,† in pharmacokinetic studies, fluorescence signals were obtained clearly at the *in vivo* tumor site (Fig. S19†), with signals remaining at above 95% of the injected dose even 9 h after injection of the therapeutic agent. NIR-Cz exhibited a circulation half-life exceeding 23.1 h (Fig. 4j). Following this, we explored the metabolic pathways of NIR-Cz by imaging the accumulation in major organs. All groups of mice were euthanized 30 hours after intratumoral injection. As depicted in Fig. S20,† at this time point, bio-distribution analysis indicates the predominant accumulation of NIR-Cz in the liver. In contrast, mice from the untreated group exhibited weak fluorescence signals in the liver. These experimental results indicate the predominant clearance of NIR-Cz through the liver.

Next, the biosafety of the theranostic agent was carefully evaluated for *in vivo* experiments. The body weight of all three groups of mice steadily increased during the treatment period, confirming that NIR-Cz exhibits negligible toxicity and side effects toward mice. Moreover, the histopathological analysis of the major organ tissues like the heart, liver, spleen, lungs and kidneys from mice in each group indicated that no damage was observed (Fig. S21†), proving that NIR-Cz could serve as a safe photothermal agent suitable for effective practical photothermal therapy.

### The combined study of channel 1 and channel 2

**Fig. 5:**
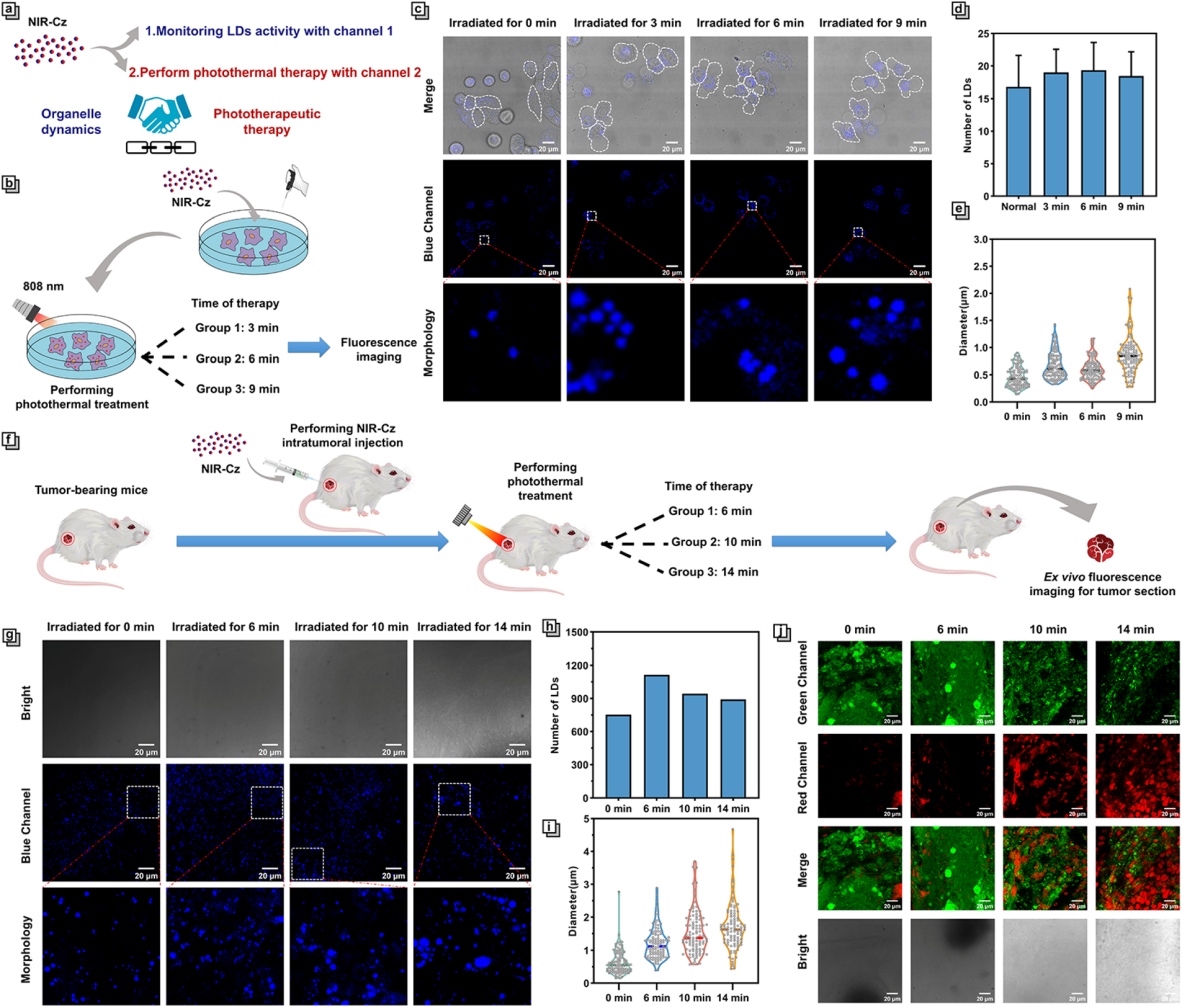
(a) Schematic illustration of the effect of combined channel 1 and channel 2. (b) Schematic illustration of the cell experimental procedure. (c) Fluorescence imaging of 4T-1 cells undergoing different treatment durations and local enlargement of cells, incubating with NIR-Cz (50 μM, *λ*_ex_ = 405 nm, and *λ*_em_ = 450–530 nm). (d) The number of LDs at different times during the PTT process. (e) The size of LDs at different times during the PTT process. (f) Schematic illustration of the *in vivo* mice experimental timeline. (g) Fluorescence imaging of tumor sections and local enlargement; tumor sections were taken from mice undergoing different treatment durations (concentration of NIR-Cz: 50 μM, *λ*_ex_ = 405 nm, and *λ*_em_ = 450–530 nm). (h) The number of LDs at different times during the PTT process *in vivo*. (i) The size of LDs at different times during the PTT process *in vivo*. (j) Calcein-AM (green) (*λ*_ex_ = 488 nm and *λ*_em_ = 500–580 nm) and propidium iodide (red) (*λ*_ex_ = 535 nm and *λ*_em_ = 600–625 nm) co-staining fluorescence images of tumor sections; tumor sections were taken from mice undergoing different treatment durations.

In recent years, photothermal therapeutic agents that target specific organelles have garnered significant attention due to their ability to leverage the close association of certain organelles with apoptotic signaling pathways.^16,17^ This allows for precise and localized heat treatment, thereby activating specific apoptotic signals more efficiently and specifically, enhancing the efficacy of PTT.^15^ However, progress in studying the dynamics of organelles during the PTT process has been slow due to the lack of multi-functional phototherapeutic agents that possess both high fluorescence quantum yield and high photothermal conversion efficiency. Thanks to the unique multifunctionality afforded by the design of NIR-Cz dual-channel excitation (Fig. 5a), we have been able to evaluate the dynamics of LDs during the PTT process.

It is reported that photothermal therapy occurs over two stages, apoptosis and necrosis; as the temperature rises, the former occurs at about 43 °C, and at temperatures above 44 °C, necrosis occurs.^29^ Therefore, to explore the underlying therapeutic mechanism of phototherapy, we focused on the apoptotic stage and performed an in-depth investigation of the LD dynamics during the process of phototherapy. Studies have reported that FSP27 (fat-specific protein of 27 kDa or Cidec) is a member of the cell death-inducing DNA fragmentation factor-α-like effector (CIDE) family, which is thought to be activators of apoptosis.^30^ It has been shown that exogenous FSP27 expression facilitates the occurrence of apoptosis, while endogenous FSP27 expression upregulation leads to lipid accumulation even in non-adipose cells,^31^ which may in turn produce more LDs. Additionally, research has indicated that lipid transfer to mitochondria and subsequent excessive accumulation can induce apoptotic processes, characterized by a reduction in lipid droplets.^32^ To provide direct evidence of lipid involvement in the process of apoptosis at the level of organelle dynamics, we leveraged the multifunctional characteristics of the therapeutic agent NIR-Cz imaging the dynamics of LDs during the PTT process. 4T-1 cells were incubated with NIR-Cz for 30 min in a cell incubator and irradiated using an 808 nm laser for a period of time until apparent apoptosis was observed (Fig. 5b). The dynamics of LDs in the cell were observed continuously *via* the blue channel every 3 min (Fig. 5c).

We found that the average quantity of LDs is correlated with the degree of apoptosis, where the quantity of LDs tends to increase first and then decrease with the progression of apoptosis (Fig. 5d). At the same time, there is no obvious change in the morphology of the LDs. In fact, the number of micro-size LDs decreases significantly, while the average diameter of LDs increases during the progression (Fig. 5e).

Subsequently, we investigated the dynamics of LDs during the PTT process *in vivo*. Tumor-bearing mice, after intratumoral injection of NIR-Cz, were divided into three groups and subjected to photothermal treatment with an 808 nm laser for varying time durations (Fig. 5f). Following treatment, high resolution spatiotemporal imaging of tissue sections was performed using confocal laser scanning microscopy (Fig. 5g). Impressively, the average number of LDs in experiments *in vivo* exhibited a similar trend to that observed in cellular experiments, increasing initially and then decreasing (Fig. 5h). The average diameter of LDs continuously increased with the progression of apoptosis (Fig. 5i). To study the correlation between the duration of treatment and the progression of apoptosis within tumor tissue, we employed calcein-AM and propidium iodide staining of tissue sections for validation, with results shown in Fig. 5j. For the non-treatment group after NIR-Cz was injected into mice, the sample predominantly exhibited a strong green fluorescence signal, with the intensity reducing steadily with the progression of apoptosis induced by increasing treatment duration. In contrast, the sample of mice from the 16 min group that received treatment with an 808 nm laser exhibited bright red fluorescence signals, revealing that the process of apoptosis within the tumor is nearing completion. Therefore, we confirmed a positive correlation between the treatment duration and the progression of apoptosis within tumor tissue.

The experiments *in vivo* have unveiled distinct experimental phenomena compared to the cellular level, such as the average diameter of LDs *in vivo* not being the same as that *in vitro*. The average diameter of LDs for the experiments *in vivo* was initially similar to that at the cellular level, measuring 0.598 μm (compared to 0.363 μm at the cellular level) before treatment, but increased to 1.563 μm (compared to 0.818 μm at the cellular level) in the later stages of treatment, which is significantly larger than the diameter of LDs in the treatment group observed in cellular experiments.

Based on our results, combining with previous research, we believe that there are two main stages during the process of phototherapy. The first stage is the accumulation of lipids. This stage is typified by an overall increase in the number of LDs and an increase in their average diameter, while the number of micro-sized LDs gradually decreases. The increase in LD numbers suggests that apoptosis induced by PTT may be influenced by the accumulation of lipids resulting from the upregulation of FSP27 expression.^33^ The second stage is the consumption of lipids, characterized by the gradual decrease in the number of intracellular lipid droplets, while the average diameter of the lipid droplets further increases. The reduction in the number of LDs may reveal that intracellular lipids support the onset of mitochondria-mediated PTT-induced apoptosis by consuming themselves.

This discovery also reveals that cell death induced by PTT is not controlled by a single signaling pathway and may be under the synergistic regulation of more than two. The underlying signaling pathways responsible for this dynamic phenomenon require further investigation. Our new findings regarding the dynamics of LDs during the PTT process support the standpoint that the mitochondria-mediated apoptotic signaling pathway, which is supported by lipids consuming themselves, may be activated later than the signaling pathway induced by the upregulation of FSP27 expression. The underlying signaling pathways responsible for this dynamic phenomenon require further investigation. All in all, to our knowledge, these are the first investigations of organelle dynamics during PTT-induced apoptosis.

## Conclusions

In conclusion, we have proposed a novel crosstalk-free multi-functional phototherapeutic agent based on a dual-channel strategy. We have also developed a proof-of-concept application during preliminary investigations of the LD dynamics during PTT. In contrast to conventional phototherapeutic agents that rely on a single-channel strategy for achieving multitasking, crosstalk-free multi-functional phototherapeutic agents feature mutually independent dual-channels. This design significantly enhances the efficiency of their respective functions, achieving a breakthrough in addressing the problem associated with multi-functional phototherapeutic agents for studying organelle dynamics during phototherapy. As such, we were able to successfully integrate organelle dynamics research with phototherapy. As a proof of concept, we developed a novel theranostic agent, NIR-Cz, which not only delivers outstanding *in vivo* therapeutic efficacy through its exceptional photothermal conversion properties at 808 nm (PCE of 35.8%) but also enables specific targeting of LDs at 405 nm, thereby enabling high spatial resolution and long-term monitoring. Furthermore, leveraging the exceptional capabilities of NIR-Cz, we have investigated the dynamics of LDs throughout the PPT process. According to our results, the LD number exhibits a non-monotonic increasing trend with variable fluctuations, characterized by an initial increase followed by a decrease. We revealed that there are two distinct stages within lipid dynamics during photothermal therapy: the accumulation and the subsequent consumption of lipids. Crosstalk-free multi-functional phototherapeutic agents based on a dual-channel strategy are expected to evolve through the continuous development and engineering of wavelengths suitable for the phototherapeutic channel and imaging channel, becoming a “one for two” phototherapeutic agent with both crosstalk-free NIR-II imaging capability and phototherapeutic capability. These advancements will allow for a multi-dimensional elucidation of the intimate relationships between organelles and apoptotic signaling pathways. Furthermore, NIR-Cz, as a therapeutic agent, is anticipated to play a significant role in biomarker research based on its potent dual-channel crosstalk-free imaging capabilities. It can be utilized to monitor the progress of photothermal therapy, identify new potential cancer therapeutic targets, and develop new combination therapeutic strategies. In conclusion, it is our belief that the connection, established by new types of multi-functional phototherapeutic agents, between organelle dynamics and photothermal processes will greatly facilitate the advancement of precisely localized treatments.

## Data availability

Data will be made available on request from Weiying Lin.

## Author contributions

Lixuan Dai: conceptualization, methodology, formal analysis, investigation, data curation, writing – original draft, writing – review & editing, and visualization. Wenxiu Li: writing – original draft and writing – review & editing. Xiaoli Zhong: formal analysis and data curation. Mingguang Ren: resources and project administration. Tony D. James: writing – review & editing. Weiying Lin: resources, writing – review & editing, supervision, and funding acquisition

## Conflicts of interest

There are no conflicts to declare.

## Supplementary Material

SC-016-D4SC08796H-s001
