# Supplementary material for: Construction of crosstalk-free multi-functional phototherapeutic agents
Source: Chem Sci. 2025 Feb 12;16(11):4775–87. doi: 10.1039/d4sc08796h (PMC11818293; doi:10.1039/d4sc08796h)
Supplement: SC-016-D4SC08796H-s001 [file SC-016-D4SC08796H-s001.pdf]

Supplementary Information (SI) for Chemical Science.
This journal is © The Royal Society of Chemistry 2025

Supporting Information for

Construction of Crosstalk-free Multi-functional Phototherapeutic Agents

Lixuan Dai[1], Wenxiu Li[1], Xiaoli Zhong[1], Mingguang Ren[4],

Tony D. James,[*, 2,3] and Weiying Lin[1, *]

[1]*Institute of Optical Materials and Chemical Biology, Guangxi Key Laboratory of Electrochemical Energy Materials, School of Chemistry and Chemical Engineering, Guangxi University, Nanning, Guangxi 530004, P. R. China*

[2] *Department of Chemistry, University of Bath, Bath, BA2 7AY, UK*

[3] *School of Chemistry and Chemical Engineering, Henan Normal University, Xinxiang 453007, P. R. China*

[4]*State Key Laboratory of Biobased Material and Green Papermaking, Key Laboratory of Pulp & Paper Science and Technology of Shandong Province/Ministry of Education, Qilu University of Technology (Shandong Academy of Sciences), Jinan 250353, P. R. China*

*Corresponding Author.

E-mail address: weiyinglin2013@163.com.

E-mail address: t.d.james@bath.ac.uk

Table

Experiments 4

Synthesis of compounds 4

Synthesis of compound 1 4

Synthesis of compound 2 5

Synthesis of compound NIR-Cz 5

Computational Details 6

In vitro experiments 6

General procedure for viscosity determination and spectral measurements 6

Calculation of the photothermal conversion efficiency 7

The Förster–Hoffmann equation 8

Calculation of fluorescence quantum yield of NIR-Cz 8

Cells incubation and imaging 8

Colocalization experiments of NIR-Cz 9

Cytotoxicity experiments 9

In vitro PTT 9

Software and methods 10

In vivo experiments 10

Animals and Tumor Model 10

In vivo antitumor efficacy 11

Table S1.. 11

Additional absorption and fluorescence spectra 12

Figure S1 12

Table S2. 12

Figure S2. 13

Figure S3. 13

Figure S4. 14

Figure S5. 14

Figure S6. 14

Figure S7. 15

Figure S8....15
Additional CLSM imaging....15
Figure S9....15
Figure S10....16
Figure S11....17
Figure S12....17
Figure S13....17
Figure S14....18
Figure S15....18
Figure S16....18
Figure S17....19
Figure S18....20
Table S3....20
Figure S19....20
Figure S20....20
Figure S21....21
NMR spectra....22
Figure S22....22
Figure S23....22
Figure S24....23
Figure S25....23
Figure S26....24
References....24

Experiments

Synthesis of compounds

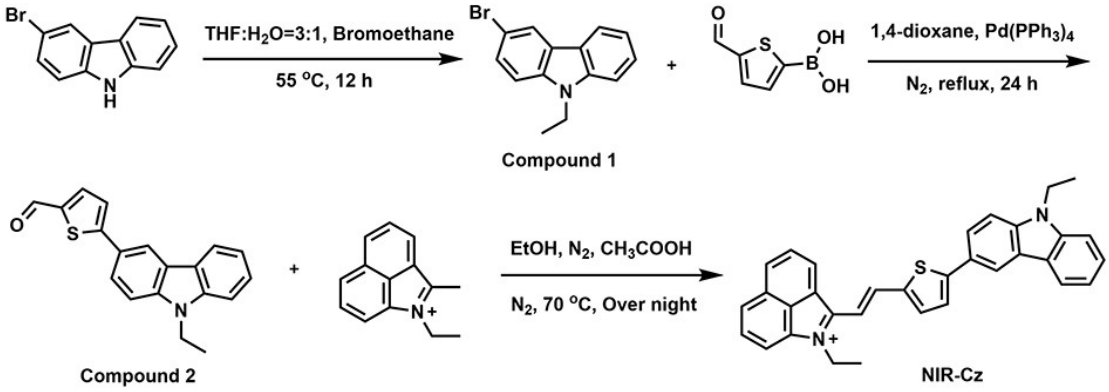

Scheme S1. Synthestic route of the fluorescent probe **NIR-Cz**.

Synthesis of compound 1

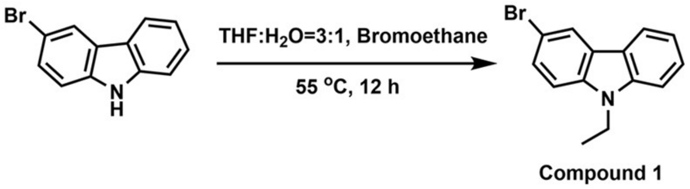

Scheme S2. Synthesis route of compound 1.

A mixture of 3-bromo-9H-carbazole (2.46 g, 10 mmol), bromoethane (6.54 g, 60 mmol), NaOH (0.80 g, 20 mmol) was added to 20 mL of a mixed THF water solvent (V_{THF} : V_{water}=3:1) and stirred in a high-pressure reactor at 60 °C for 12 h. After the reaction was completed as determined by thin layer chromatography (TLC) analysis, the reaction system was cooled to room temperature and then purified by column chromatography to obtain a white solid, compound 1 (1.45g, 5.3 mmol), Yield: 53 %. ^{1}H NMR (400 MHz, DMSO-d_6) δ 8.40 (s, 1H), 8.21 (d, J = 7.8 Hz, 1H), 7.65 – 7.53 (m, 3H), 7.49 (t, J = 7.6 Hz, 1H), 7.21 (t, J = 7.4 Hz, 1H), 4.43 (q, J = 7.0 Hz, 2H), 1.29 (t, J = 7.1 Hz, 3H).

Synthesis of compound 2

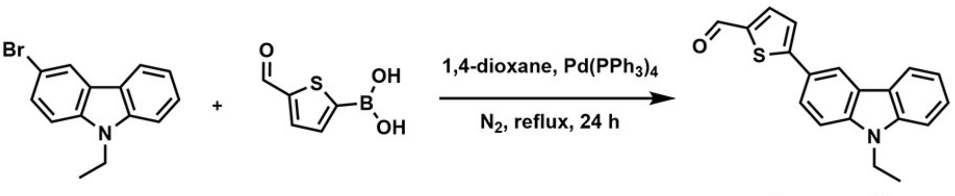

Scheme S3. Synthesis route of the compound 2.

A mixture of compound 1 (0.73 g, 2.66 mmol), 5-formyl-2-thiopheneboronic acid (0.50 g, 3.19 mmol), K_2CO_3 (1.11 g, 8 mmol), $Pd(PPh_3)_4$ was added to 20 mL of a mixed THF water solvent (V_{THF} : V_{water}=3:1) in a round bottom flask under nitrogen, and the mixture was stirred at 60 °C for 12 h. After the reaction was completed as determined by TLC analysis, the reaction system was cooled to room temperature, extracted by DCM, keep organic layer and dried, and then purified by column chromatography to obtain a pale-yellow solid, compound 2 (0.28 g,0.93 mmol), Yield: 35 %. ^{1}H NMR (400 MHz, Chloroform-*d*) δ 9.90 (s, 1H), 8.43 (s, 1H), 8.17 (d, *J* = 7.7 Hz, 1H), 7.83 – 7.77 (m, 2H), 7.54 (t, *J* = 7.7 Hz, 1H), 7.46 (d, *J* = 12.7 Hz, 3H), 7.31 (t, *J* = 7.6 Hz, 1H), 4.41 (q, *J* = 7.2 Hz, 2H), 1.48 (t, *J* = 7.2 Hz, 3H).

Synthesis of compound NIR-Cz

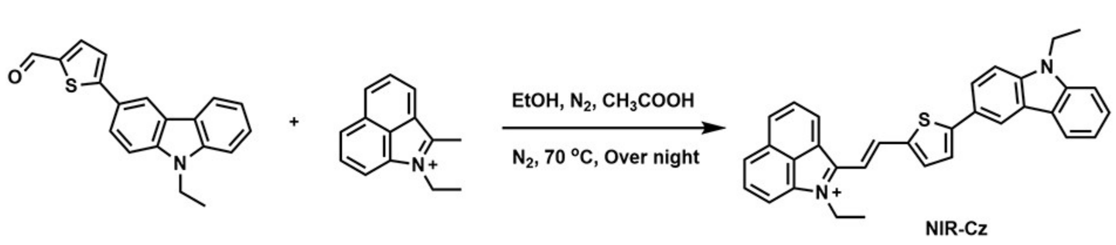

Scheme S3. Synthesis route of the fluorescent probe **NIR-Cz**.

A mixture of compound 2 (0.10 g, 0.32 mmol), 1-ethyl-2-ethylbenzo[cd]indol-1-ium (0.12 g, 0.65 mmol), glacial acetic acid (2 drop), and 20 mL EtOH were added to a round bottom flask under nitrogen, and the mixture was stirred at 70 °C over night. After the reaction was completed as determined by TLC analysis, the reaction system was cooled to room temperature, extracted by DCM, keep organic layer and dried, and

then purified by column chromatography to obtain a dark-blue solid, **NIR-Cz** (0.10 g, 0.22 mmol), Yield: 70 %.^{1}H NMR (400 MHz, DMSO-d_6) δ 9.27 (d, *J* = 7.5 Hz, 1H), 9.07 (d, *J* = 15.4 Hz, 1H), 8.72 (s, 1H), 8.64 (d, *J* = 8.0 Hz, 1H), 8.29 – 8.23 (m, 4H), 8.14 (t, *J* = 7.7 Hz, 1H), 7.99 – 7.87 (m, 3H), 7.74 (d, *J* = 8.6 Hz, 1H), 7.67 (d, *J* = 8.2 Hz, 1H), 7.60 – 7.49 (m, 2H), 7.29 (t, *J* = 7.4 Hz, 1H), 4.80 (q, *J* = 6.8 Hz, 2H), 4.48 (q, *J* = 6.7 Hz, 2H), 1.53 (t, *J* = 7.2 Hz, 3H), 1.34 (t, *J* = 7.1 Hz, 3H). Calculated for $C_{33}H_{27}N_2S^+$, [M-I^-] 483.1889, found: 483.1889.

Computational Details

Clog *P* values of **NIR-Cz** and other commercial probes calculated by Chemdraw, XLOGP3 and Molinspiration.

Chemdraw: Clog *P* value of compounds can be calculated in "Chemical Properties Window".

XLOGP3[1]: Clog *P* value of compounds can be calculated on the website for free (http://www.sioc-ccbg.ac.cn/skins/ccbgwebsite/software/xlogp3/).

Molinspiration: Clog *P* value of compounds can be calculated on the website for free (https://molinspiration.com/cgi-bin/properties).

HOMO/LUMO energies were performed by the Gaussian 16 program. The geometry optimization of ground states was computed with density functional theory (DFT) at the B3LYP/6-311G levels.

In vitro experiments

General procedure for viscosity determination and spectral measurements

The solvents were acquired by mixing PBS-glycerol systems in different proportions. Each viscosity value was recorded using a NDJ-9s rotational viscometer.

The stock solution of **NIR-Cz** (5 mM) was prepared in DMSO. The test solution contained **NIR-Cz** (10.0 μM), PBS-glycerol in different proportions. These solutions were sonicated for 5 min to remove air bubbles. After standing at room temperature for 1 h, the sample was measured in a UV spectrophotometer and a fluorescence spectrophotometer.

Calculation of the photothermal conversion efficiency

The photothermal conversion efficiency (η) of **NIR-Cz** was calculated according to previous reports. The calculation was carried out as following equations[2]:

$$\eta = \frac{hs(T_{Max} - T_{surr}) - Q_{Dis}}{I(1 - 10^{-A_{\lambda}})} \quad (1)$$

where η is the photothermal conversion efficiency of **NIR-Cz** which means the ratio of absorbed light energy converting to thermal energy, and h is the heat transfer coefficient, s is the surface area of the container, Q_{Dis} represents heat dissipated from the laser mediated by the solvent and container, I is the laser power, A_{λ} is the absorbance of **NIR-Cz** at the wavelength of 808 nm in water.

$$hs = \frac{mC_{solvent}}{\tau_s} \quad (2)$$

where m is the mass of the solution containing the **NIR-Cz**, C is the specific heat capacity of the solvent (C_{water} = $4.2 \bullet 10^3$ J/(kg•°C)), and τs is the associated time constant.

$$t = -\tau_s \ln(\theta) \quad (3)$$

Next introduce θ, which is defined as dimensionless parameter, known as the driving force temperature.

$$\theta = \frac{T - T_{surr}}{T_{Max} - T_{surr}} \quad (4)$$

T_{max} and T_{Surr} are the maximum steady state temperature and the environmental temperature, respectively.

The photothermal effect and photothermal conversion efficiency of **NIR-Cz** was

conducted by using a 808 nm laser for irradiation.

The Förster–Hoffmann equation

The Forster–Hoffmann equation s1 was utilized to correlate the relationship between the fluorescence emission intensity of **NI-VD** and the value of solvent viscosity.

$$\log I = C + x\log \eta \quad (5)$$

$$\log \tau = C + x\log \eta \quad (6)$$

Where η is the value of viscosity, I is the emission intensity, τ is the fluorescence lifetime, C is a constant, and x represents the sensitivity of the fluorescent probe to viscosity.

Calculation of fluorescence quantum yield of NIR-Cz

The fluorescence quantum yield of the compounds was obtained by the following equation

$$\Phi_s = \Phi_r \left(\frac{A_r\lambda_r}{A_s\lambda_s}\right)\left(\frac{{n_s}^2}{{n_r}^2}\right)\frac{F_s}{F_r} \quad (7)$$

s and *r* are the sample and the reference values, respectively. Fluorescence quantum yield is *Φ*; The integrated intensity expressed by *F*; *A* and *n* stand for the absorbance and refractive index respectively.

Cells incubation and imaging

Human hepatocellular liver carcinoma cells (HeLa cells), Mouse breast cancer cells (4T-1 cells), were cultured in Dulbecco's Modified Eagle's medium (DMEM) containing 10% fetal bovine serum (FBS) and 1% penicillin-streptomycin at 37 ° C in a humidified atmosphere composed of 5% CO_2. The cells were maintained in an exponential growth phase by periodic sub-cultivation. Before any cell experiments were performed, multi-functional phototherapeutic agent **NIR-Cz** (stock solution of **NIR-Cz**, 10 mM in DMSO) was added to the medium, the volume of DMSO in the medium was controlled to be about 1%. Before imaging, the live cells were incubated with this medium for different periods, then washed with PBS three times.

Colocalization experiments of NIR-Cz

Before imaging, the cells were cultured in 35 mm culture dishes (Nest, Wuxi, China) for 24 h. After incubated with commercial dyes with **NIR-Cz** 30 min, cells were washed with PBS for three times and visualized by confocal microscopy. Excited and detection was performed in a sequential mode under the following conditions: **NIR-Cz** (10 μM) with fluorescent dyes were excited with a 405 nm laser, detected in the range from 450 to 530 nm; The commercial dyes of Subcellular organelles including BODIPY 493/503 (Beyotime, Shanghai, China.) (10 μM, λ_{ex} = 488 nm, λ_{em}: 498 - 530 nm) and Mito-Tracker Deep Red FM (Beyotime, Shanghai, China.) (2 μM, λ_{ex} = 644 nm, λ_{em}: 654 - 690 nm) scale bar: 20 μm.

Cytotoxicity experiments

HeLa and 4T-1 cells were plated at 96-well plates (1× 10^4 cells per well and incubated in 100 μL) for 24 h, then the cells were treated with **NIR-Cz** at various concentrations. 30 min later, these cells were exposed to 808 nm laser irradiation (500 mW/cm^{-2}) for 5 min. Meanwhile, **NIR-Cz** incubated cells without laser irradiation under the same experimental conditions were also used for the dark cytotoxicity study. After further incubation for 18 h, MTT solution (100 μL of 0.5 mg/mL in DMEM) was added to each well, and the cells were further incubated at 37 ºC for 4 h. Subsequently, the medium was carefully removed, and 100 μL DMSO was added to dissolve the formazan crystals. The absorbance value was measured at 490 nm with a microplate reader (Bio-Rad, USA) and the cell viability was calculated:

$$Cell\ viability\ (\%) = \frac{OD_{Drug} - OD_{Blank}}{OD_{Control} - OD_{Blank}} \times 100\% \quad (8)$$

All of the measurements were performed five times and the values are presented as the mean ± SD.

***In vitro* PTT**

4T-1 cells were seeded on 35 mm confocal dishes and allowed to stabilize for 24

h. The cells were then treated with 1 mL multi-functional phototherapeutic agent **NIR-Cz** (stock solution of **NIR-Cz**, 50 mM in DMSO). After 30 min incubation, the cells were irradiated with a 808 nm laser (500 mW/cm^2) for 15 min to induce photothermal cytotoxicity. The mode of cell death was examined by using Calcein AM/ propidium iodide (PI) staining kit. Excited and detection was performed in a sequential mode under the following conditions: **NIR-Cz** with fluorescent dyes were excited with a 405 nm laser, detected in the range from 450 to 530 nm; Calcein AM (Beyotime, Shanghai, China.) (λ_{ex} = 488 nm, λ_{em}: 500 - 580 nm) and PI (Beyotime, Shanghai, China.) (2 μM, λ_{ex} = 535 nm, λ_{em}: 600 - 625 nm) scale bar: 100, 50 and 20 μm.

Software and methods

Unless otherwise stated, the fluorescent traces from hand-segmented ROI from at least three fields of view from at least two images were extracted in Image J Fiji, and Pearson's coefficient corrections were derived using the instrument software LAS X. The power and sample size were calculated with two-sample t-Test using the Origin 2019b.

In vivo experiments

Animals and Tumor Model

Five-week-old Female BALB/c mice (license number: SCXK 2019-0010) were purchased from SiPeiFu Biotechnology (Beijing, China), and were housed under aseptic conditions in small animal isolators with free access to food and water. All animal experiments were reviewed and approved by the Animal Care and Experiment Committee of Guangxi University. To establish 4T-1 tumor bearing BALB/c mice, 4T-1 cell suspensions (1 × 10^6 cells) were subcutaneously injected into the flank of BALB/c mice depilated. The tumor volume of 4T-1 tumor bearing mice was calculated:

$$volume\ A = a \times b^2/2 \qquad (9)$$

a and *b* are length and width respectively. After the tumor volumes were about 70 mm^3, mice were used for photothermal therapy.

In vivo antitumor efficacy

The mice were divided into 3 groups (n = 3) when the tumor reached 70 mm^3. (1) Intratumor injection of PBS, then irradiated by 808 nm laser irradiation (500 mW/cm^2) only once during the process of photothermal therapy, irradiation time was 15 min (50 μL) (control group). (2) Intratumor injection of **NIR-Cz** (50 μM, 50 μL) (+ **NIR-Cz** group). (3) Intratumor injection of **NIR-Cz**, then irradiated by 808 nm laser irradiation (500 mW/cm^2) only once during the process of photothermal therapy, irradiation time was 15 min (50 μM, 50 μL) (50 μM, 50 μL) (+**NIR-Cz** and +Light group). The body weight and tumor volume were monitored every 2 days for 14 days. After treatment, all mice were sacrificed and, the tumors were collected and weighed. Major organs (heart, liver, spleen, lung, kidney) and tumor were examined by hematoxylin and eosin (H&E) staining and observed by a fluorescence microscope.

Table S1. The combined imaging modality in several multifunctional phototherapeutic agents. (λ_{ex}: Excitation wavelength).

Multifunctional phototherapeutic agent	Treating λ_{ex} (nm)	Imaging λ_{ex} (nm)	Combined imaging modality	Refs.
**FA-LSC NPs**	808	808	NIR-II *in vivo* imaging	Chem. Sci., 2024, **15**, 10969-10979.
**HBCP NPs**	655	635	FL/PA *in vivo* dual-modal imaging	Biosens.Bioelectron., **2022**, 216, 114612.
**DTTVBI NPs**	808	/	NIR-II *in vivo* imaging	J. Am. Chem. Soc., **2023**, 145, 334–344
**RDM-BDP**	660	635	Subcellular FL and NIR-I *in vivo* imaging	J. Am. Chem. Soc., **2018**, 140, 15820–15826
**IR-FE-FC@DSPE-S-S-PEG**	808	808	NIR-II *in vivo* imaging	Chem. Eng. J., **2023**, 463, 142372.
**2IB**	White light	405	Super-resolution imaging	Sci. China. Chem., **2022**, 65, 2528–2537.

**NIR-Cz**	808	405	Subcellular dynamics imaging	This work

Additional absorption and fluorescence spectra

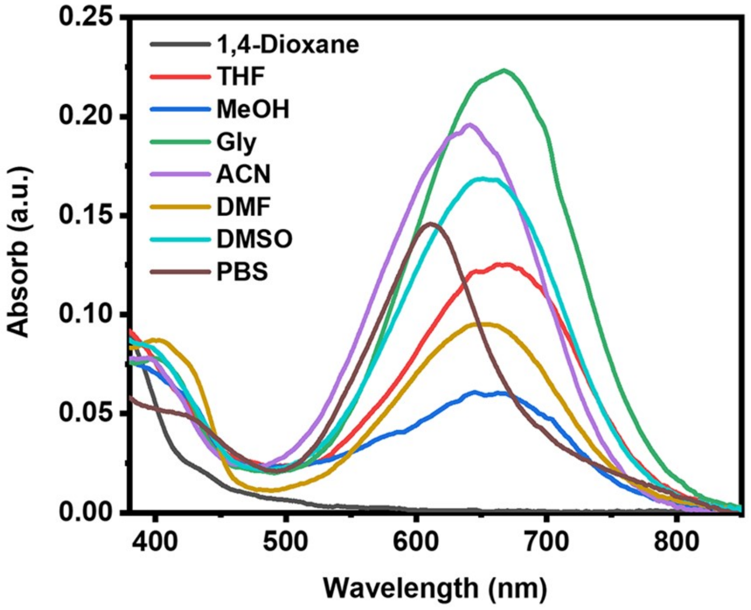

Figure S1. UV - vis absorption spectra of **NIR-Cz** in various solvents.

Solvent	λ_{Abs}(nm)	$\lambda_{Abs}/\lambda_{Em}$(nm)	Stokes shift (nm)	Φ (%)
MeOH	406	530	124	56.1
Gly	408	542	134	27.3
ACN	405	492	87	83.3
DMSO	405	511	106	97.2
PBS	424	477	53	0.9

Table S2. Summary of the photophysical properties of channel 1 of **NIR-Cz** in some solvents. (λ_{Abs}, absorption at λ_{max}; λ_{Em} emission at λ_{max}; φ, quantum yield.)

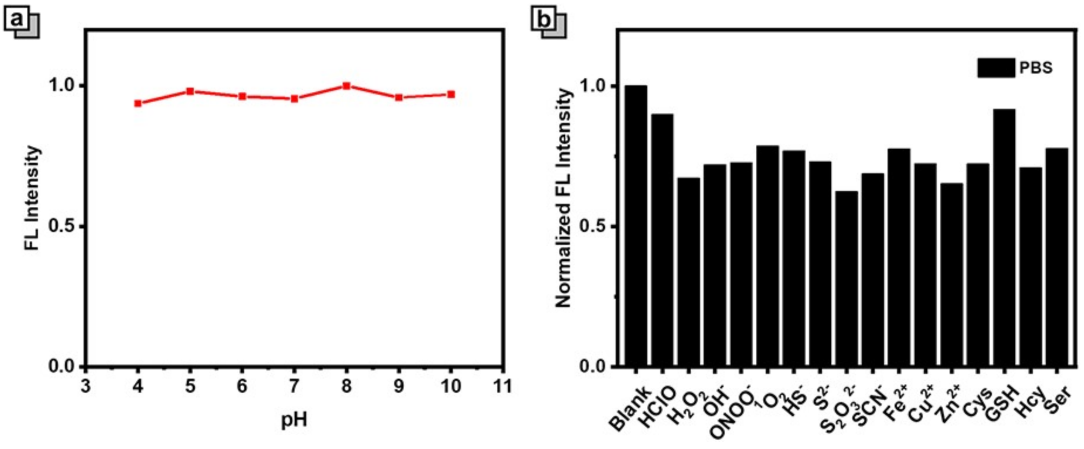

Figure S2. a. Normalized fluorescence intensity of **NIR-Cz** in PBS solution with different pH, b. Normalized fluorescence intensity of **NIR-Cz** in PBS solution (20 mM, pH 7.4) with various species (100 mM). (λ_{ex} = 405 nm).

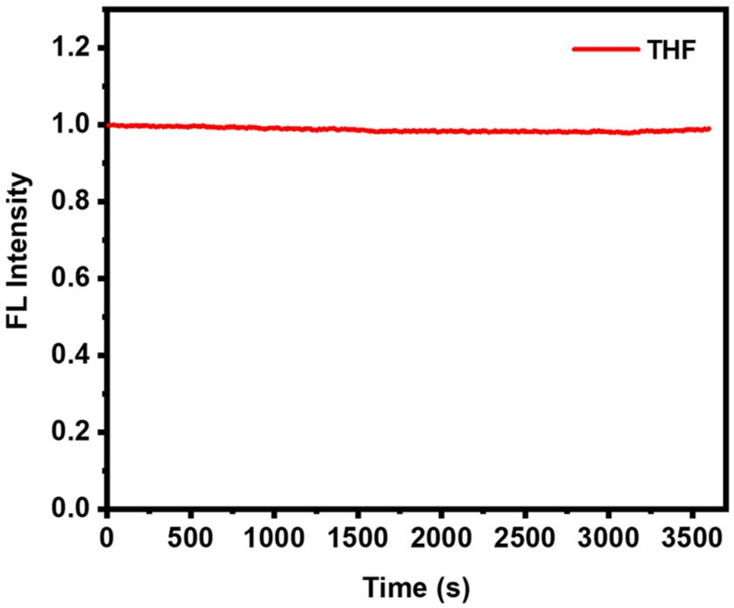

Figure S3. Photostability of probe **NIR-Cz** in THF (λ_{ex} = 405 nm).

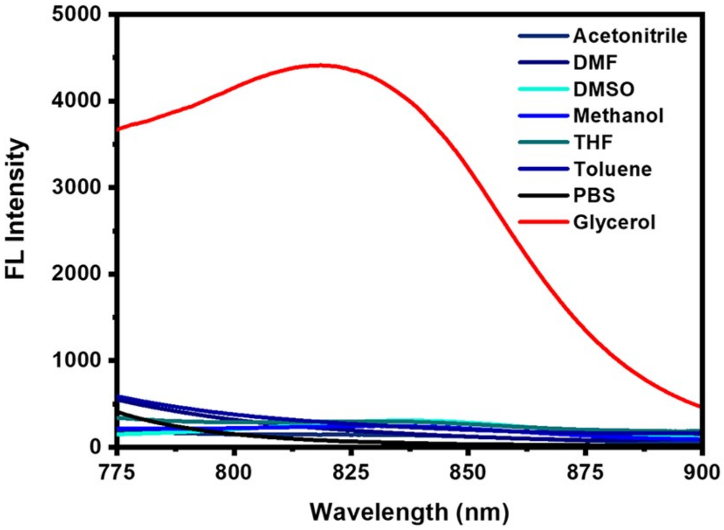

Figure S4. Fluorescence emission spectra of **NIR-Cz** in various solvents (λ_{ex} = 690 nm).

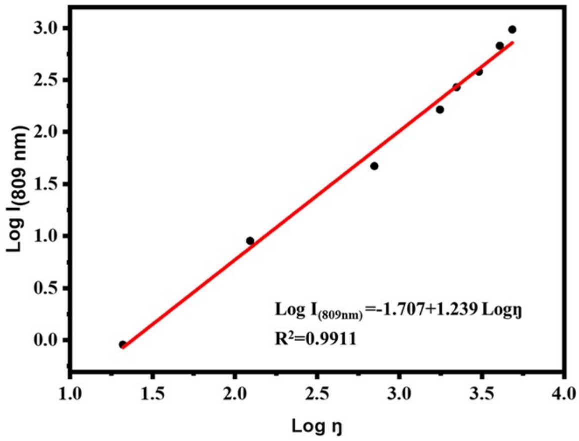

Figure S5. Linear relationship (R^2 = 0.9911) between log $I_{(809\ nm)}$ and log ŋ ranging from 3.46–965 cP.

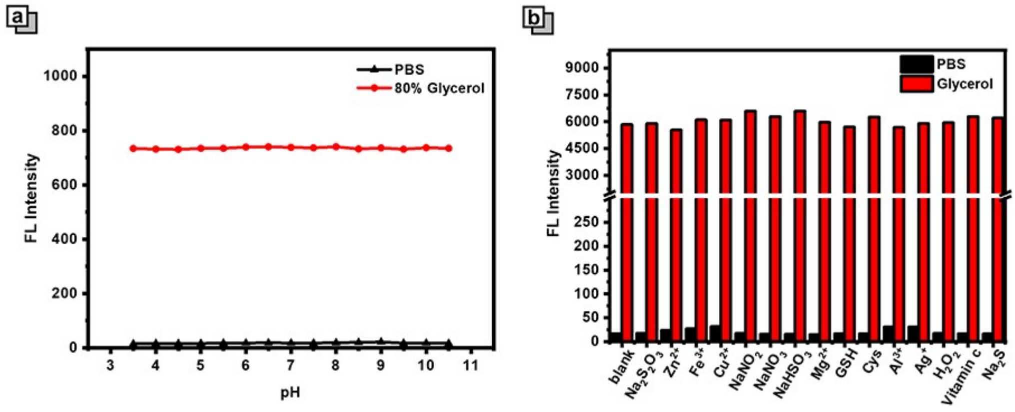

Figure S6. a. Fluorescence intensity of **NIR-Cz** in PBS solution with different pH, b.

Fluorescence intensity of **NIR-Cz** in PBS solution (20 mM, pH 7.4) with various species (100 mM). (λ_{ex} = 690 nm).

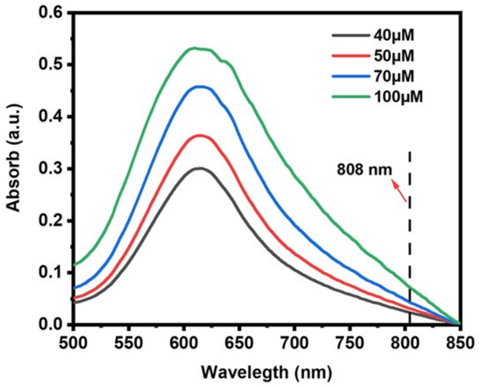

Figure S7. UV - vis absorption spectra of different concentrations **NIR-Cz** in PBS solution.

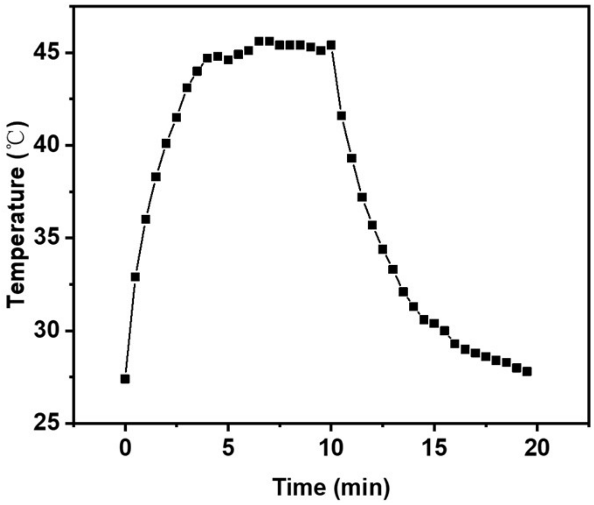

Figure S8. ΔT response to 808 nm laser on and off in period of 1200 s.

Additional CLSM imaging

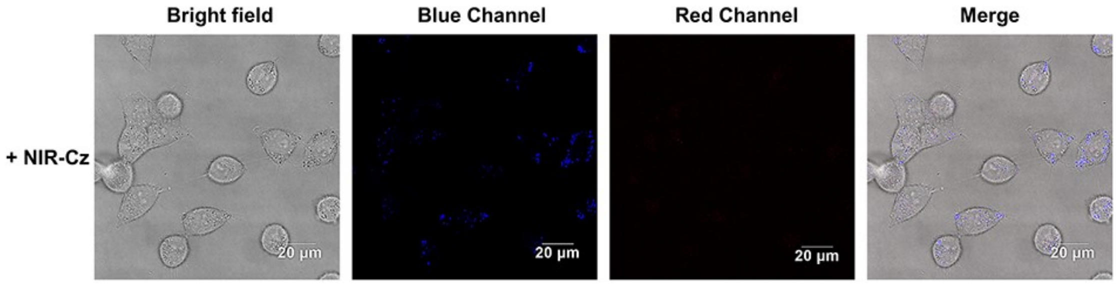

Figure S9. Fluorescence images of HeLa cells incubated with only **NIR-Cz**. (50 μM, λex = 405 nm, λem: 450-530 nm)

Commercial dye	Structural formula	Chemdraw	XLOGP3	Molinspiration
Nonyl Acridine Orange	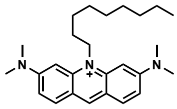	3.90	7.80	9.64
Janus Green B	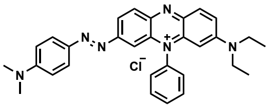	4.11	7.61	9.67
MitoTracker Deep Red FM	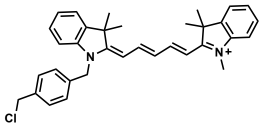	7.32	8.17	5.26
MitoTracker Orange FM	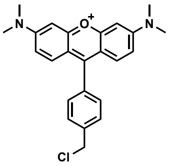	7.39	5.70	3.15
LipiDye ® II	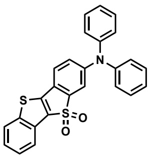	7.69	6.71	7.25
LipiDye Lipid Droplet Green	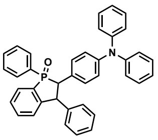	8.31	8.75	8.93
BODIPY 740/752	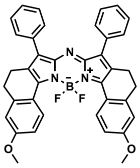	9.52	8.90	9.14
**NIR-Cz**	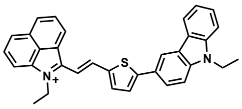	10.58	8.05	9.92

Table S3 The Clog*P* values of **NIR-Cz** and several commercial dyes calculated by different methodologies.

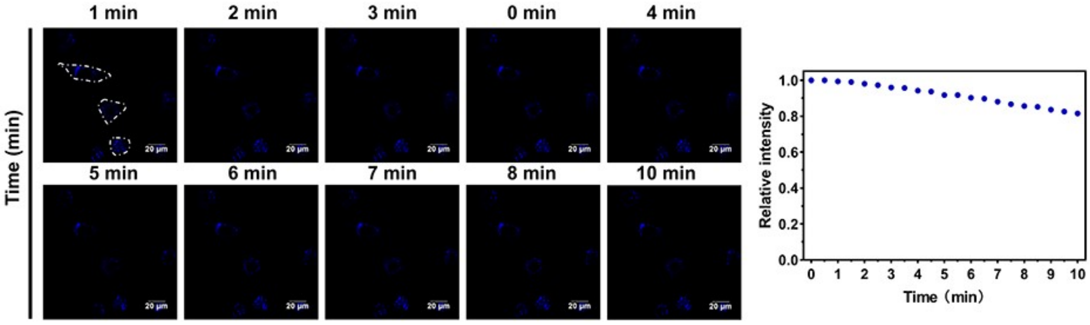

Figure S10. The photostability of **NIR-Cz** during the imaging. (50 μM, λ_{ex} = 405 nm, λ_{em}: 450-530 nm)

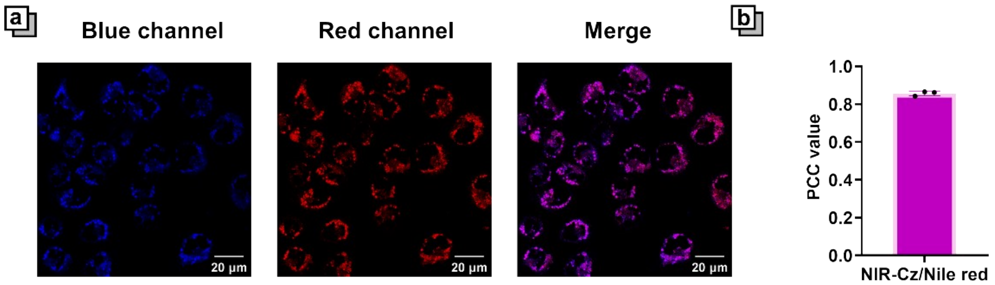

Figure S11. a. Confocal fluorescence images of HeLa cells co-stained with **NIR-Cz** (10 μM, λex = 405 nm, λem: 450-530 nm) and **Nile red** (10 μM, λex = 561 nm, λem: 584-630 nm). b. and the quantitative analysis for the co-localization of **NIR-Cz** and commercial dyes.

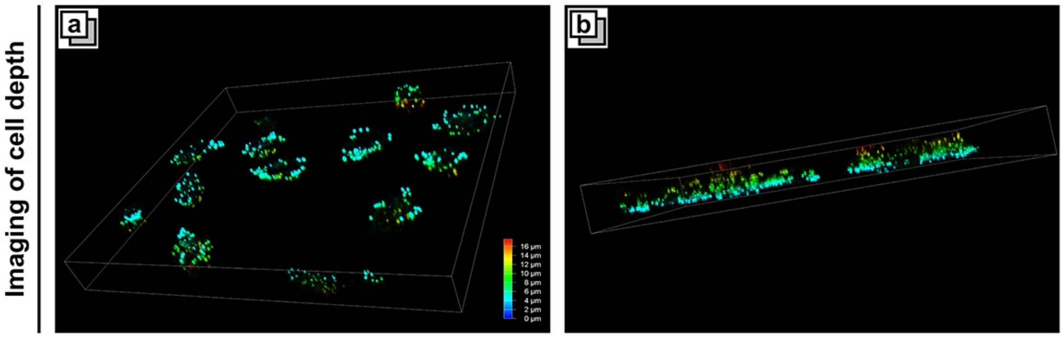

Figure S12. a. Front and b. side view of confocal fluorescence images of HeLa cell depth stained with **NIR-Cz**, in order from low to high expression, the depth displays dark blue, light blue, green, yellow, and red (10 μM, λex = 405 nm, λem: 450-530 nm).

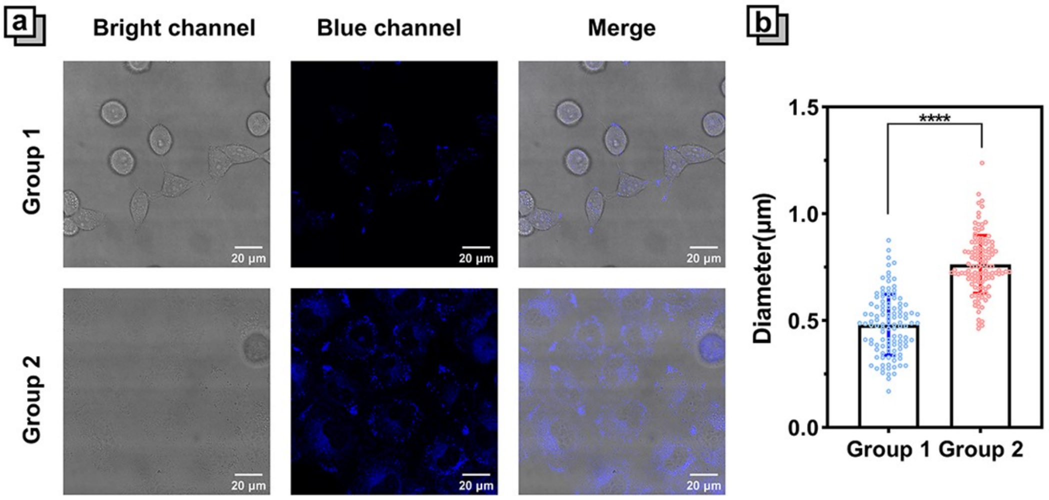

Figure S13. a. Confocal fluorescence images of two groups of HeLa cells, Group 1: cells are directly treated with **NIR-Cz**, Group 2: cells are pre-incubated with OA (200 μM) for 4 hours before treated with **NIR-Cz**. (10 μM, λex = 405 nm, λem: 450-530 nm).

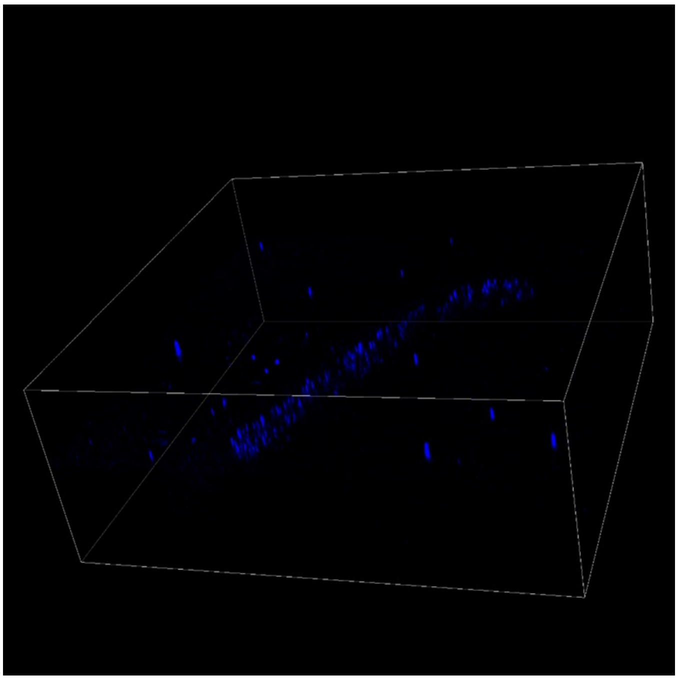

Figure S14. Front view of confocal fluorescence images of *C. elegans* in 3D model stained with **NIR-Cz** (10 μM, λex = 405 nm, λem: 450-530 nm).

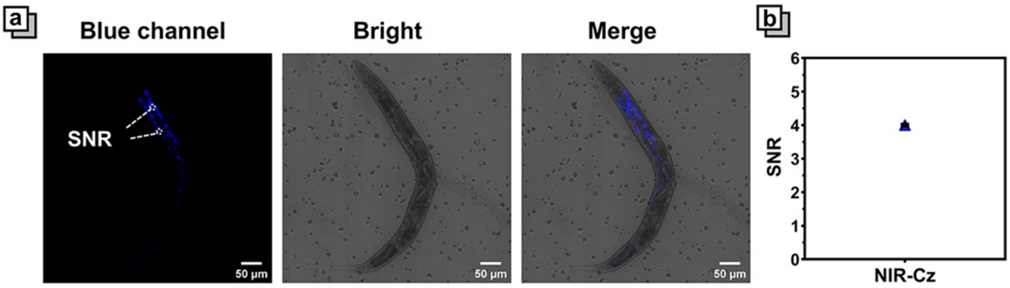

Figure S15. a. Confocal fluorescence images of *C. elegans* staining with **NIR-Cz** (10 μM, λex = 405 nm, λem: 450-530 nm). b. The SNR value of **NIR-Cz** in imaging of *C. elegans*.

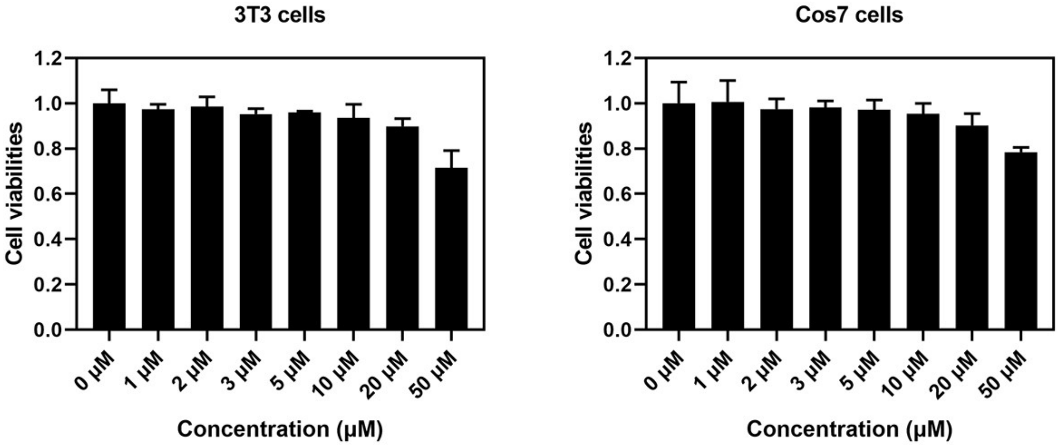

Figure S16. Cell viability results of the 3T3 cells and Cos7 cells stained with **NIR-Cz**

24 h by MTT assay. The results are expressed as percentages of the dye-free controls.

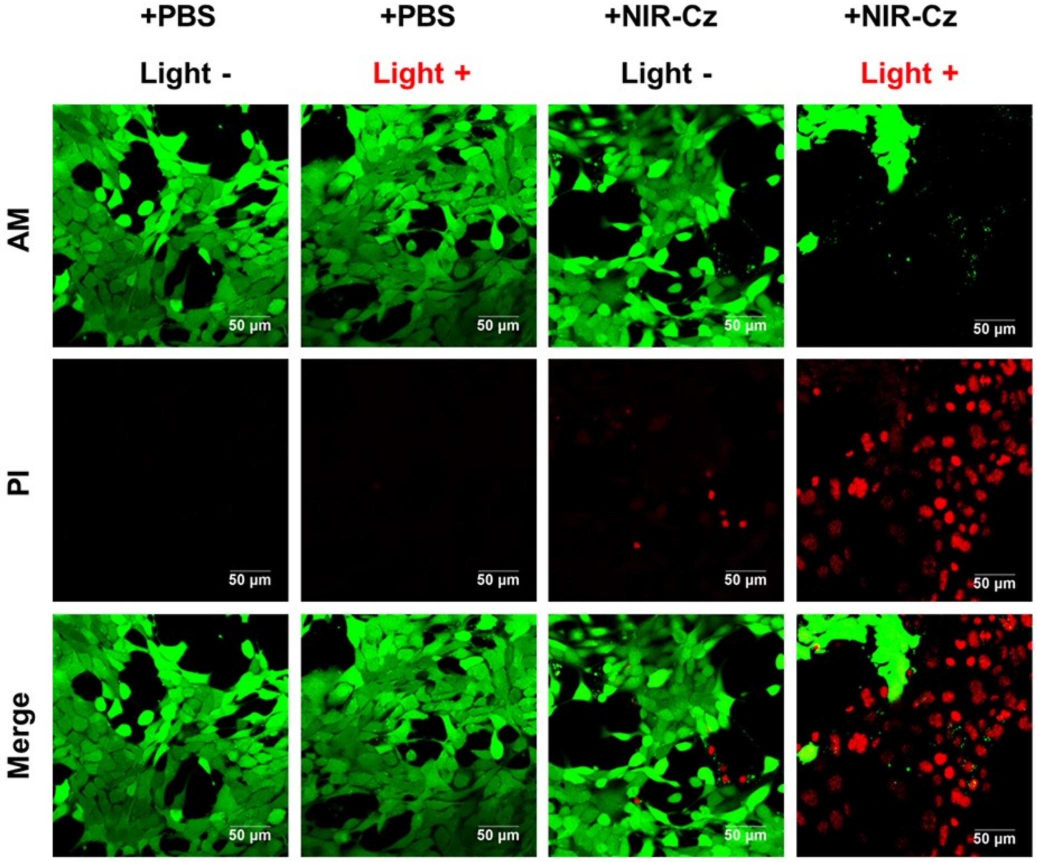

Figure S17. Calcein AM (green) (λ_{ex} = 488 nm, λ_{em}: 500-580 nm) and propidium iodide (red) (λ_{ex} = 535 nm, λ_{em}: 600-625 nm) co-staining fluorescence images of 4T-1 cells after different treatments. 808 nm laser irradiation (500 mW/cm^2, 5 min) was conducted after cells were incubated with **NIR-Cz** (50 μM). Scale bar: 50 μm.

Additional phototherapy imaging

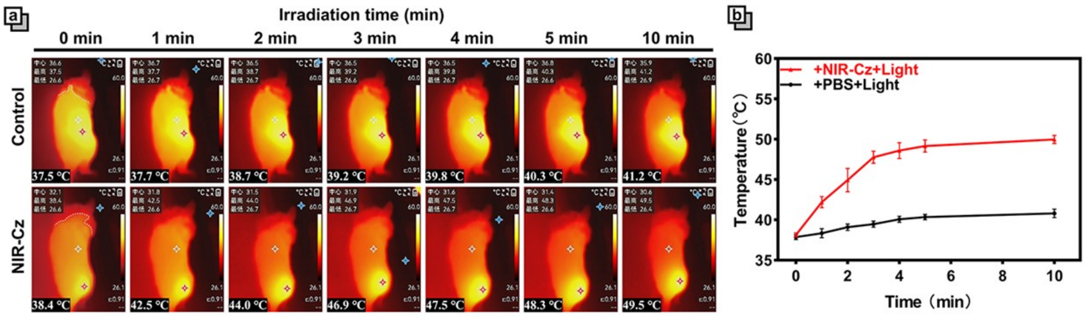

Figure S18. a. Thermal IR images of 4T1 tumor-bearing mice after intratumoral injection of **NIR-Cz** or PBS and exposure to 808 nm laser irradiation (500 mW/cm^2) for 10 min. The dashed line in first picture per group represents the contour line between the dorsal fur and skin, serving as a feature to distinguish different mice. b. Local tumor area temperature images of the mice at the 10 min of NIR irradiation.

Alpha	Sample Size	Power
0.05	3	1
0.05	5	1
0.05	7	1
0.05	11	1

Table S3. The power calculation of sample size in tumor therapy experiment.

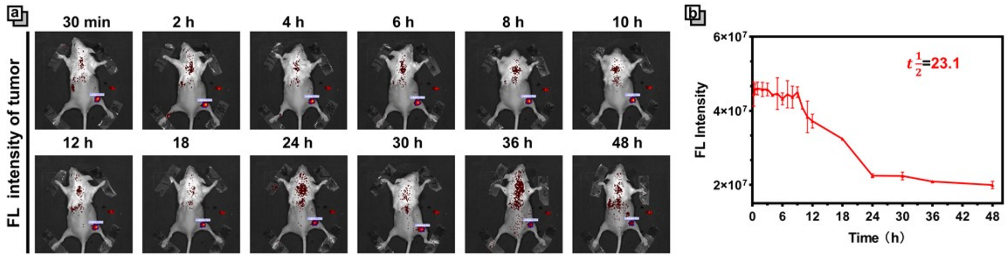

Figure S19. a. NIR fluorescence images of the mice at different monitoring times after intratumoral injection of **NIR-Cz**. b. Fluorescence intensity in tumors from mice post-administration of **NIR-Cz** at various time intervals.

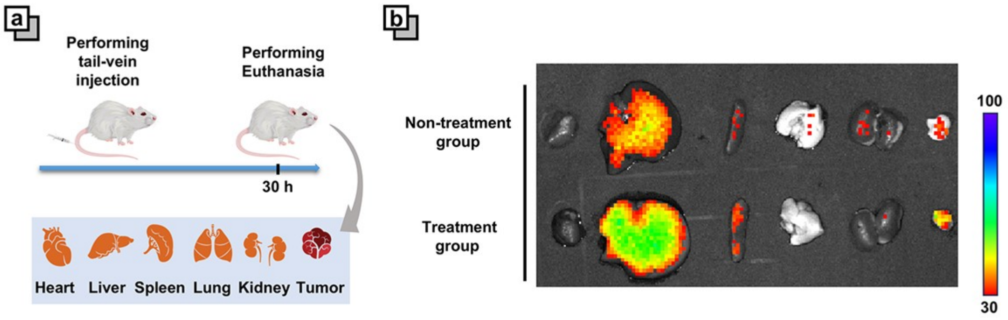

Figure S20. a. Schematic flow of the experiment. b. *Ex vivo* fluorescence images of the tumor and major organs of different groups of mice at 30 h after intratumoral injection of **NIR-Cz**. Non-treatment group: intratumoral injection of PBS; treatment group: intratumoral injection of **NIR-Cz**

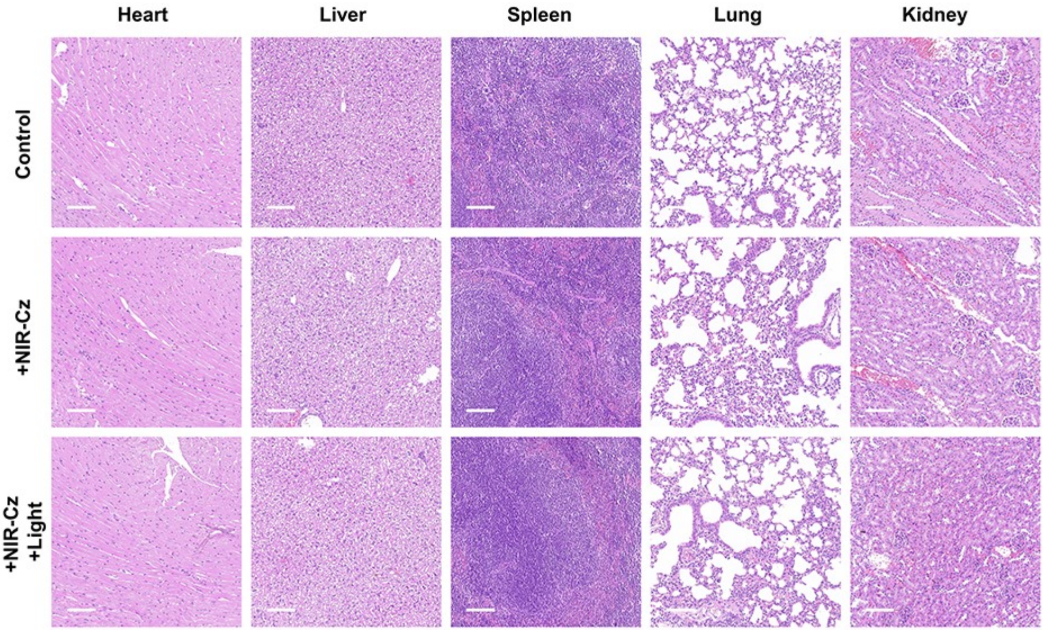

Figure S21. H&E staining images of major organs of 4T-1 tumor bearing mice in different treatment groups. Scale bars: 100 μm.

NMR spectra

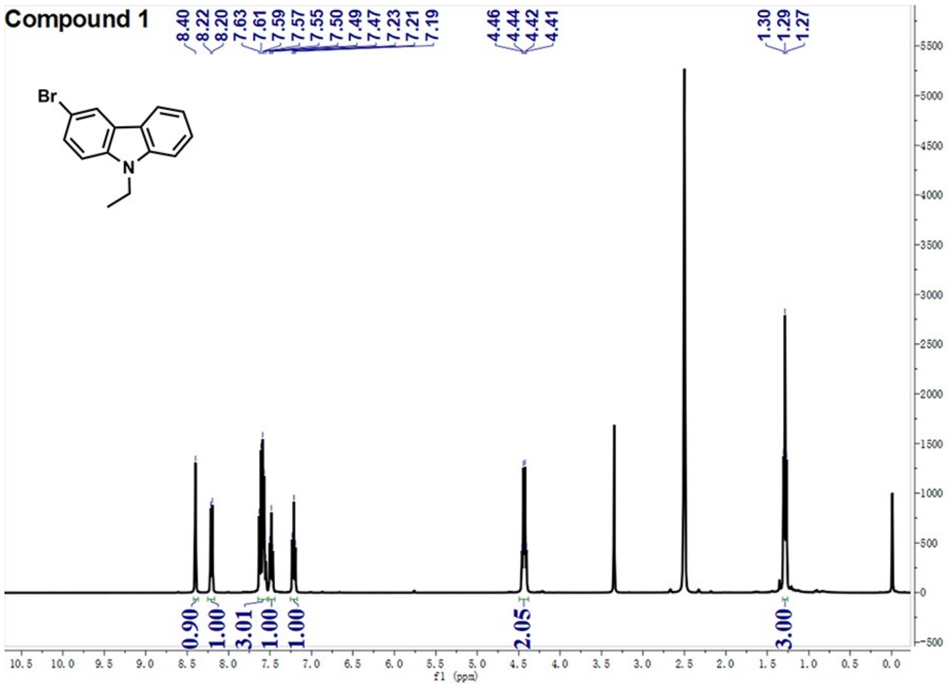

Figure S22. The ^{1}H NMR spectrum of compound 1 in d_6-DMSO.

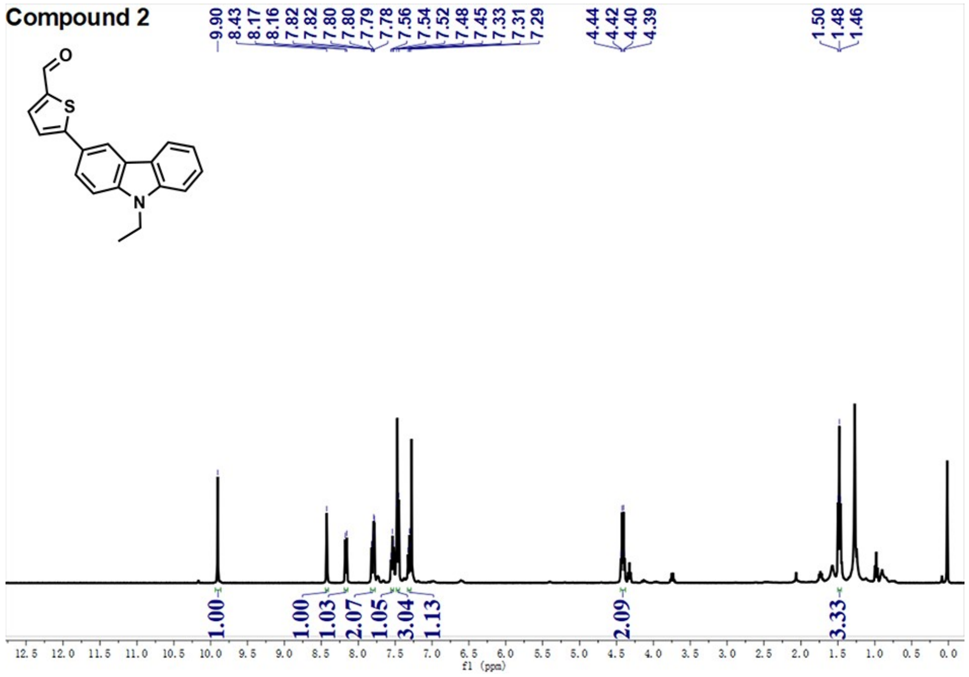

Figure S23. The ^{1}H NMR spectrum of compound 2 in Chloroform-*d*.

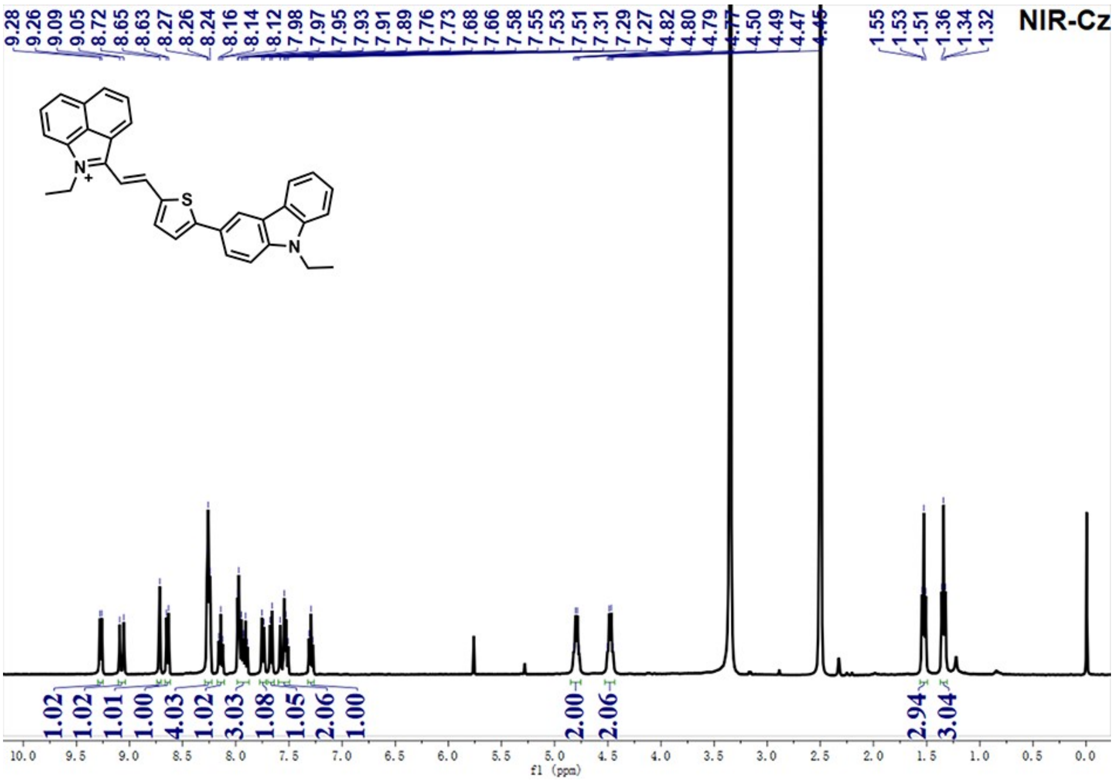

Figure S24. The ^{1}H NMR spectrum of **NIR-Cz** in d_6-DMSO.

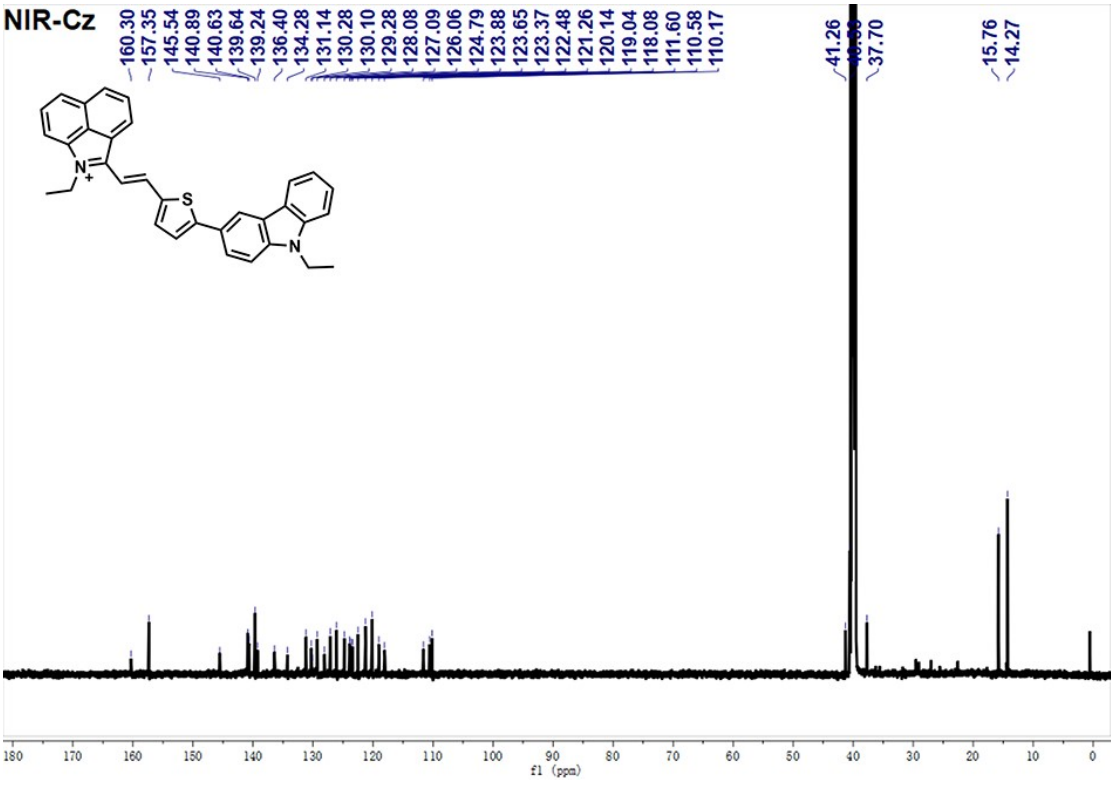

Figure S25. The ^{13}C NMR spectrum of **NIR-Cz** in d_6-DMSO.

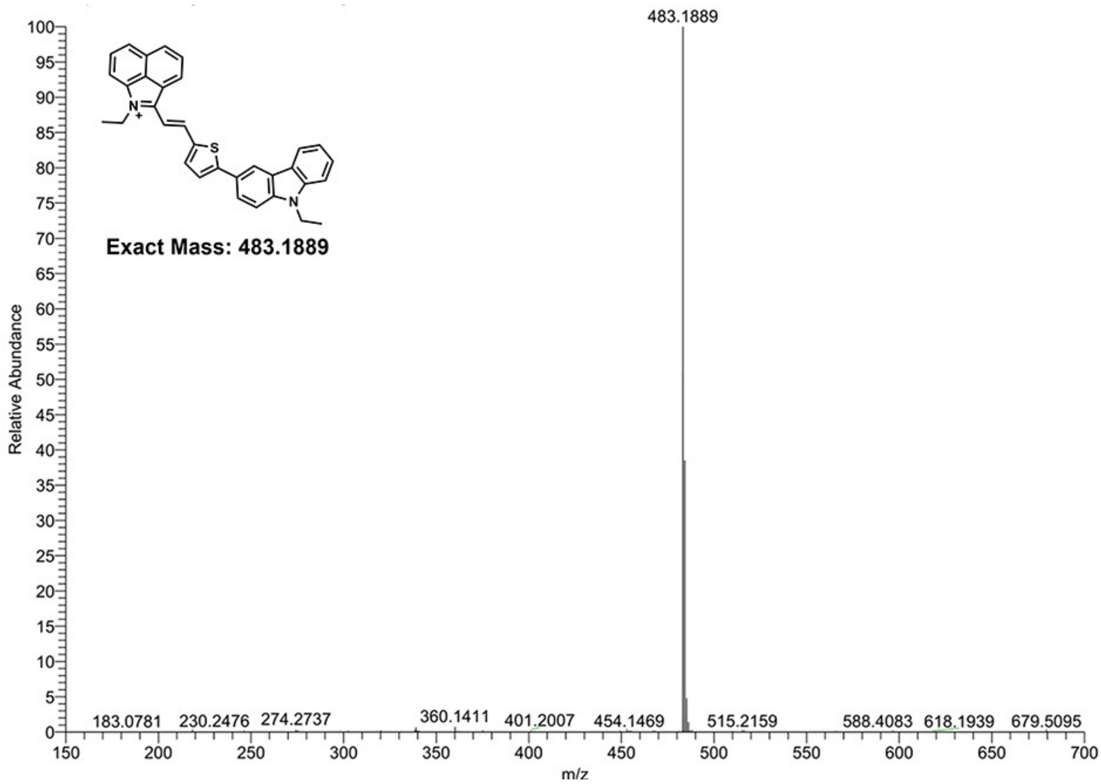

Figure S26. HRMS spectrum of probe **NIR-Cz** in MeOH ([M-H]- calcd: 483.1889 Found： 483.1889).

References

[1] T. Cheng, Y. Zhao, X. Li, F. Lin, Y. Xu, X. Zhang, Y. Li, R. Wang, L. Lai, Computation of Octanol−Water Partition Coefficients by Guiding an Additive Model with Knowledge, J. Chem Inf. Model. 6 (2007) 2140-2148.

[2] W. Ren, Y. Yan, L. Zeng, Z. Shi, A. Gong, P. Schaaf, D. Wang, J. Zhao, B. Zou, H. Yu, G. Chen, E.M.B. Brown, A. Wu, A Near Infrared Light Triggered Hydrogenated Black TiO2 for Cancer Photothermal Therapy, Adv. Healthc. Mater. 10 (2015) 1526-1536.
